# Competence and control beliefs in three cultures: a German personality test applied at home, in Kenya and Poland

**DOI:** 10.1186/s40359-025-03416-2

**Published:** 2025-09-10

**Authors:** Michaela Heinecke-Müller, Julia Miczka, Josephine N. Arasa, Claudia Quaiser-Pohl

**Affiliations:** 1 Institute of Psychology, University of Koblenz, Universitätsstraße 1, 56070 Koblenz, Germany; 2 Department of Psychology, United States International University of Nairobi, Nairobi, Kenya

**Keywords:** Competence and control beliefs, Self-efficacy, Equivalence, Personality, Cross-cultural

## Abstract

**Background:**

Competence and control beliefs are core self-evaluations with increasing value as predictors (e.g., in clinical, organizational, environmental, and educational psychology), and they are assumed to have a universal core that is shared across cultures. The Inventory for Self-Efficacy and Externality (I-SEE, Greve et al., 10.1207/S1532706XID0104_02), which has already been translated and transferred from Germany to several cultures, has not yet been analyzed with regard to its cross-cultural applicability. While a recently updated German short form shows promise for transcultural application, further research into the culturally specific meaning of the construct is needed.

**Methods:**

A total of 1,084 participants from Germany, Kenya, and Poland completed a 32-item personality test designed to assess self-efficacy (self-concept of own competence and internality) and externality (social and fatalistic). The factor structures were compared via exploratory factor analysis (EFA) and structural equation modeling (ESEM).

**Results:**

Two factors emerged consistently and altogether represented the content of internal and external control beliefs. The theoretically proposed measurement model, which identified four distinct factors, was not replicated. Inconsistencies between the subsamples were evident in the areas of control related to others, chance and religiosity. Structural equivalence could only be partially supported, whereas metric and scalar equivalence were not confirmed.

**Conclusions:**

The intra- and intercultural usability of the measure are questioned, while potential advancements are given with the social and fatalistic aspects of the underlying construct. Given the greater significance of these control beliefs in non-Western cultures, a more detailed examination of their allocation (internal, external, or both) is warranted. An embedment in a more comprehensive, transculturally applicable personality theory is discussed.

**Supplementary Information:**

The online version contains supplementary material available at 10.1186/s40359-025-03416-2.

## Background

The well-established personality questionnaire I-SEE [1; German: FKK, 2] assesses competence and control beliefs with four independent primary factors. As a fine-grained scale—although less efficient than unidimensional scales—it allows an analytical view of socioemotional and cognitive details of the construct. Translated and transferred to several cultures worldwide in diverse application fields (e.g., clinical, work, environmental, and educational psychology), a thorough analysis of culture-specific processes that might form different aspects of control beliefs is still lacking. The allocation of certain items and the construction of the scale essentially reflects a typical W.E.I.R.D. (Germanic) worldview rooted in a pre-Reunification Western German context. The validity of this approach is called into question here.

In the last several decades, *invariance* or *equivalence testing* of psychological instruments has been extensively discussed [3, 4]. The core issue is as follows: Would it be appropriate to apply an instrument across diverse fields of application, groups of persons with specific characteristics, near or distant cultures, and different time periods [5]? Only a few studies report on the intermediate results of the lengthy and costly work processes that, at best, lead to improved instruments that have proven to be invariant [6].

The aim of this study is to explore the factorial characteristics of the I-SEE measure in the originating country (Germany) compared with samples from divergent cultures—one quite distant (Kenya) and the other neighboring (Poland). It is expected, that both “at home and abroad” adjustments of the instrument are advisable for a variety of reasons. Most notably, the test’s norms have aged and are restricted to Germany. Next, history has led to changes in the “global village”, such as the emerging perception that threats such as pandemics or war-like events are common challenges to humanity. Finally, there are culture-specific aspects to investigate.

Carefully considering that society is diverse and constantly evolving, even within delimited regions or populations, the aim of this study is not to deliver a ready-made instrument but rather to offer an insight into an ongoing investigation. The current focus is on the structure equivalence of the I-SEE concept, which has travelled over time and across cultural borders from 1990s Germany to the present day, to Poland and Kenya. The conclusions drawn will inform further research into the adaptation of the measure. Insight is provided into the details of the following analyses conducted: explorative factor analyses and exploratory structural equation modeling (ESEM). Data from Germany, Kenya, and Poland are compared. Building on a multimethod exploration of content and factorial validity, the study’s results serve as a basis for transcultural adaptations of the measure in the future.

### Competence and control beliefs are nested in culture

Psychological control refers to certain forces that may lead to consequences: Is a goal attainable with one’s own competence and effort, are others in power, or is it rather a question of chance or fate? Control influences any process of action, learning, development, adaptation, success, well-being, or even health recovery [7]. In contrast to power- or helplessness [8], terms addressing psychological control became renowned internationally with Rotter’s locus of control [9] and Bandura’s self-efficacy [10]. Currently, several overworked personality theories and measures from this construct family are mostly assembled under core self-evaluations [11].

Control beliefs are highly valued predictors, even compared with broader constructs (e.g., the Big Five) [12], because they feature only a medium degree of abstraction. An area-specific competence belief was found to implicate a gain in performance of up to 28% [13], positive task and social experiences, and increased job motivation [12]. Experiencing self-efficacy and control over one’s life is closely related to well-being and optimism in stress appraisal over a whole life span [14, 15]. A sense of control prevents illness and promotes recovery [16].

**Fig. 1:**
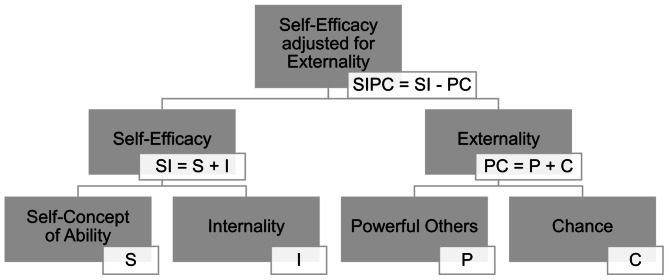
Conceptual structure of the I-SEE measure according to Krampen [1, 2]: primary, secondary, and tertiary scales

This report refers to competence and control beliefs as defined by Krampen’s action-theoretical model [17, 18], for which the I-SEE scale is offered as a measure [1, 2]. It is composed of four relatively independent personality aspects expressing generalized expectations (see Fig. 1): two aspects feed self-efficacy (an expectation of being capable of achieving self-propelling results), and two other aspects sum up to externality (e.g., influence exerted by others or fate). These *internal*^1^ and *external* secondary scales are designed to oppose each other, so, optionally, one is subtracted from the other at a tertiary level.

Research has focused on finding a cross-culturally shared core of a unidimensional control aspect: self-efficacy or internality [15, 19–21]. Undoubtedly, humans share an existential need for control. However, the idea that other people, chance or fate are outside of, and even opposed to, the individual’s sense of self is not necessarily shared across cultures [2, 22–24]. For example, if certain cultures differ in the way they live out social relations and religion, their beliefs about competence and control beliefs may also differ in terms of their composition and how they function.

A globalized lifestyle has been spreading throughout metropolitan areas worldwide [25]. Nevertheless, sociocultural contexts shape the way people perceive, think and act [26–28]. Research on universal personality features is invaluable for understanding human functioning. However, more often than not, an unavailability of *emic* instruments (as being culture-specific “from the inside”) leads to an overemphasis of *etic* approaches, those being foreign measures transferred to other cultures and contexts. A robust transcultural approach aims “[…] to describe a construct or theory with an integrated or balanced treatment of universal and culture-specific aspects.” (Cheung et al. [29], p. 597). This *emic-etic* perspective should apply to all levels of measurement equivalence, inevitably beginning with the level of items, and their content validity.

The results of the latest research indicate that a balance must be found between the extremes of specificity and universality [30, 31]. When the I-SEE measure is used to predict typical outcomes relating to competence and control beliefs (e.g., learning motivation, job performance and well-being), samples from Germany and Kenya resemble each other quite closely, although they do not exactly match. Additional qualitative data on item comprehension showed that, when assessed in Kenya, certain items tended to shift from external to internal. By contrast with German responses, powerful others (e.g., professionals, family members, politicians) are not considered to be as emotionally negative. They do not necessarily impede the individual but can also be viewed as an internal resource. For example, relatives can provide support or raise self-worth as part of the extended self.

This report focuses on three cultural regions: Germany (the measure’s country of origin), Kenya, and Poland. This selection is derived from an ongoing research collaboration that provided convenience samples. For the latter two countries, virtually no research data are available on the subject.

The German culture is roughly characterized as individualistic and secularized and as a typical W.E.I.R.D. country [28, 32]. After German reunification, intracultural differences in control orientations were clearly detectable between the Eastern and Western regions [33, 34]. Therefore, decades later, the German personality of today is likely not the same as it was when the I-SEE measure was developed at that time [35–37].

In Kenya, the social group is of relatively greater importance than in other societies: African countries are broadly recognized as being relatively collectivistic [32], even considering that data on this enormous and highly diverse continent are still patchy and incomplete [38, 39]. The specific background of Kenya’s *Ubuntu* culture [40, 41] creates social-relational accents that tend to disagree with the W.E.I.R.D. perspective [42, 43]. It has been proven to impact commercial and business behavior [44], as well as the climate and attitudes in organizations [45–47]. The consequences for psychological control are still only scarcely explored.

Although Germany’s and Kenya’s two distant cultures have shared some important relations over several decades [48], Poland has shared Germany’s borders and an intertwined cultural history for centuries. Consequently, most cultural markers between Poland and Germany resemble each other quite well (e.g., the degree of individualism expressed) [32]. However, in Poland, 84% of the population declare themselves to be Roman Catholic believers [49]. Compared with other secularized European countries, Poland still retains a high degree of religiousness, which shapes values and relations in everyday life [50, 51]. In this country, specific features are therefore expected considering beliefs about the influence of others or fate.

This study examines how well the I-SEE measure aligns with its theoretical background when applied in three different cultural regions (Germany, Kenya, and Poland). If a shared core of the concept is clearly detectable, groundwork is laid for an assessment of the measure’s factorial invariance. This involves testing whether the theoretically proposed structure can be applied across the included samples. This is achieved by evaluating the fit of the model under the additional constraints of equal factor loadings (i.e., scale intervals are equal) and intercepts (i.e., latent means can be compared) for the involved groups. Although a common core may be clearly discernible, the structure of the measure is expected to differ significantly between the three samples. If this is the case, an exploratory analysis at the level of items is conducted to determine which item content differs most between the samples and whether any still represent a common core.

## Method

### Sample structure

A total of 1,084 surveys were conducted in Germany, Kenya, and Poland between 2017 and 2020 (before the COVID-19 pandemic; see Table 1). This convenience sample consisted of 8% Kenyan, 10% Polish, and 82% German test participants. The German share consists of a student sample and a large mixed sample, so a population-representative subsample could be drawn with respect to age, sex, employment, relationship status, education and migration background. All the subjects were informed adequately, took part voluntarily, and were recruited from the surroundings of large cities’ universities.

**Table 1:** Sample structure

		Sample 1 (*n* = 52)	Sample 2 (*n* = 833)	Sample 2.a (*n* = 206)	Sample 3 (*n* = 91)	Sample 4 (*n* = 108)	Total 5 (*N* = 1,084)
Age mean (*SD*)		25 (8.3)	33 (16)	47 (17.6)	29 (9.8)	41 (19.2)	32 (13.3)
Sex ^a^	Male Female	18 34	284 505	101 105	36 55	40 67	378 661
Country		Germany	Germany	Germany	Kenya	Poland	all
Recruitment		Campus	Internet/ snowball sampling	Drawn from Sample 2	Campus/ snowball sampling	Campus/ snowball sampling	mixed
Participants / Reward		University students and employees / course credit or none	University students and general population / course credit, individual feedback	Represen-tative / see above	University students and middle manage-ment employees / 5 US $	University students / none	mixed
Year		2017	2020	2020	2017–2018	2018	2017–2020

Note. Cases in which numbers do not add up are due to missing data

^a^ Other sexes were not specified

Representativeness could not be realized for the non-German part at this stage of research. The Kenyan sample comprises a total of 16 different identifications (20% Kikuyu, 11% African, 9% Luo, 8% Kamba, […]), which reflects the country’s high level of cultural diversity. Moreover, the sample is remarkably older than the Kenyan population as a whole. In contrast, the Polish sample is younger, as Poland has a similar age structure to Germany. The gender percentages could not be controlled for. Overall, these small pilot samples do not allow the results to be generalized to the populations of countries or cultures, and they also limit the interpretation of measurement invariance testing. Nevertheless, the sample provides a basis for a stepwise exploration of factor replicability: Germany today is compared with norms from the 1990s, with one sample from a distant (Kenya) and one from a neighboring country (Poland).

### The questionnaire

Most of the surveys were conducted using paper‒pencil questionnaires. Parts of the German samples answered via a web-based version. The questionnaire was presented in German, English (Kenyan sample), or Polish [52]. In Kenya, approximately 70 regional languages are spoken, with Kiswahili and English being the official languages, so an English version seems to provide the highest compatibility.

The entire questionnaire lasted approximately 30 min and consisted of three sections: 1. *Competence and control beliefs*. The competence and control beliefs questionnaire I-SEE [1, 2] consists of four intercorrelating, but nonetheless distinctive scales: self-concept of ability (S), internal control orientation (I), chance control orientation (C), and powerful others control orientation (P; see Fig. 1). The manual provides reliability estimates (Cronbach’s α) ranging from 0.70 (I) to 0.89 (PC), test‒retest reliability ranging from 0.68 (P) to 0.87 (PC) within a three-month period, and profile reliability of 0.53 (primary scales) and 0.66 (secondary scales; *N* = 2028 [2]).

2. *Demographic items*. Gender, age, (sub)culture, and education were recorded. 3. *Other variables*. Depending on the subsample, several typical outcome variables were supplemented but are not reported here (e.g., well-being and threat perceptibility).

### Data analyses

Internal consistency was assessed for the FKK’s primary scales (S, I, P and C) via McDonald’s ω [53] (see Table 2). Cronbach’s alpha is added to allow for comparison. Cronbach’s Alpha values roughly correspond with the norm sample’s given in the manual [2]. For Kenya, internal consistency scores are relatively lower, as expected. For complete item means, standard deviations, and corrected item‒total correlations of each sample, see Table A1 in the Supplemental Materials. Correlations were computed via IBM SPSS Statistics for Windows 29.0. Wherever preconditions for parametric methods were violated, nonparametric estimators were reported instead. Table 3 displays the scale intercorrelations for each sample as well as the data from the instrument`s manual. Most coefficients approximate the norm sample’s values in magnitude and direction. A divergent pattern emerges in the relationship of the internal and external primary scales. The overall tendency across subsamples is that correlations between the internal S/I scales and the external P/C scales are lower than those in the norm sample, often approaching zero.

First, explorative factor analyses (EFA) were conducted for the I-SEE scale in the transnational sample. Separate EFAs for each of the subsamples then served to evaluate and compare the resulting factor solutions, looking for culture-specific patterns as well as transcultural commonalities. Notably, the measure was not designed on a factor analytical basis. Despite this fact, Krampen [2] reported on explorative factor analyses in the manual to substantiate the theoretical framework, allowing for comparison of actual data with the norm sample. Following recommendations from Eid et al. [54], the principal axis analysis approach was chosen. Items with insufficient reliability are omitted in advance for each of the samples (see Supplementals).

**Table 2:** Means (M), standard deviations (SD), and internal consistency (Cronbach’s α and McDonald’s ω) of the I-SEE scales

Scale^a^		Sample						
		Norm^a^	1	2	2.a	3	4	5
S	*M*	31.9	32.37	31.87	33.18	33.03	29.77	31.78
	*SD*	6.12	5.61	7.83	5.71	5.75	6.25	7.46
	α	0.76	0.77	0.81	0.76	0.54	0.74	0.79
	ω	-	0.73	0.81	0.73	0.62	0.73	0.73
	(ω)	-	0.78	0.83	0.75	0.65	0.73	0.76
I	*M*	32.4	32.33	32.74	33.79	34.90	34.88	33.11
	*SD*	5.44	4.84	6.52	4.77	5.87	4.86	6.30
	α	0.70	0.69	0.73	0.70	0.59	0.67	0.71
	ω	-	0.67	0.74	0.68	0.58	0.66	0.69
	(ω)	-	0.70	0.79	0.71	0.62	0.69	0.71
*P*	*M*	26.1	23.17	22.68	26.22	17.07	22.89	22.25
	*SD*	5.89	4.54	6.05	5.20	5.63	5.97	6.14
	α	0.73	0.69	0.76	0.67	0.68	0.72	0.75
	ω	-	0.66	0.76	0.69	0.67		0.66
	(ω)	-	0.70	0.76	0.69	0.69		0.67
C	*M*	26.8	25.02	23.67	24.48	19.10	26.64	23.74
	*SD*	6.24	6.61	6.92	6.33	6.58	6.30	7.05
	α	0.75	0.81	0.76	0.79	0.70	0.71	0.76
	ω	-	0.82	0.75	0.78	0.69	0.72	0.78
	(ω)	-	0.82	0.75	0.79	0.70	0.72	0.78
SI	*M*	64.2	64.69	64.61	67.01	68.23	64.49	64.90
	*SD*	10.3	8.68	13.17	8.97	10.14	9.53	12.46
	α	0.83	0.79	0.86	0.80	0.72	0.80	0.84
	ω	-	0.79		0.80	0.80	0.78	0.78
	(ω)	-	0.81		0.81	0.81	0.79	0.80
PC	*M*	53.0	48.19	46.36	50.73	36.10	50.29	45.97
	*SD*	10.8	9.96	11.88	10.00	10.92	11.17	12.07
	α	0.83	0.84	0.85	0.81	0.81	0.82	0.85
	ω	-	0.85	0.85	0.82	0.79	0.84	0.81
	(ω)	-	0.86	0.85	0.82	0.79	0.85	0.81
SI-PC	*M*	11.3	16.50	18.15	16.10	32.10	14.21	18.87
	*SD*	18.2	16.85	16.21	15.45	17.56	17.28	16.98
	α	0.89	0.89	0.77	0.84	0.82	0.85	0.80
	ω	-	0.89		0.84	0.82	0.89	
	(ω)	-	0.89		0.85	0.81	0.90	

Note. In parentheses: McDonald’s Omega if poor item dropped

Samples: 1 (German students and university employees; *n* = 52), 2 (German snowball sample; *n* = 833), 2.a (German representative sample; *n* = 206), 3 (Kenya; *n* = 91), 4 (Poland; *n* = 108), 5 (transcultural total sample; *n* = 1,084)

Primary scales: S (Self-concept of ability; I (Internality); P (Powerful others); C (Chance)

Secondary scales: SI (S + I = Self-efficacy); PC (*P* + C = Externality)

Tertiary scale: SI-PC (SI – PC = Self-efficacy adjusted for externality)

^a^ Krampen [2]

**Table 3:** I-SEE scale intercorrelations

Scale	Sample	I	*P*	C	SI	PC	SIPC
S	1	0.38^*^	− 0.46^*^	− 0.54^*^	0.86^*^	− 0.57^*^	0.77^*^
	2	0.67^*^	0.12^*^	0.02	0.93^*^	0.08	0.71^*^
	2.a	0.44^*^	− 0.27^*^	− 0.38^*^	0.88^*^	− 0.39^*^	0.78^*^
	3	0.61^*^	− 0.38^*^	− 0.44^*^	0.90^*^	− 0.47^*^	0.82^*^
	4	0.49^*^	− 0.49^*^	− 0.43^*^	0.90^*^	− 0.49^*^	0.82^*^
	5	0.63^*^	0.01	− 0.09^*^	0.92^*^	− 0.04	0.72^*^
	Norm ^a^	0.56^*^	− 0.47^*^	− 0.51^*^	0.90^*^	− 0.55^*^	0.83^*^
I	1	-	− 0.33	− 0.51^*^	0.80^*^	− 0.48^*^	0.69^*^
	2		0.28^*^	0.18^*^	0.90^*^	0.26	0.54^*^
	2.a		− 0.13	− 0.03	0.82^*^	− 0.07	0.52^*^
	3		− 0.10	− 0.20	0.89^*^	− 0.17	0.65^*^
	4		− 0.21	− 0.15	0.83^*^	− 0.20	0.59^*^
	5		0.17^*^	0.10^*^	0.89^*^	0.15^*^	0.55^*^
	Norm ^a^		− 0.30^*^	− 0.26^*^	0.87^*^	− 0.32^*^	0.68^*^
*P*	1		-	0.59^*^	− 0.48^*^	0.86^*^	− 0.76^*^
	2			0.66^*^	0.22^*^	0.90^*^	− 0.48^*^
	2.a			0.48^*^	− 0.25^*^	0.83^*^	− 0.70^*^
	3			0.50^*^	− 0.28^*^	0.88^*^	− 0.71^*^
	4			0.68^*^	− 0.42^*^	0.91^*^	− 0.80^*^
	5			0.66^*^	0.09^*^	0.90^*^	− 0.57^*^
	Norm ^a^			0.57^*^	− 0.45^*^	0.88^*^	− 0.77^*^
C	1			-	− 0.63^*^	0.92^*^	− 0.87^*^
	2				0.11^*^	0.92^*^	− 0.58^*^
	2.a				− 0.26^*^	0.89^*^	− 0.73^*^
	3				− 0.38^*^	0.91^*^	− 0.79^*^
	4				− 0.36^*^	0.92^*^	− 0.77^*^
	5				0.00	0.92^*^	− 0.65^*^
	Norm ^a^				− 0.44^*^	0.89^*^	− 0.78^*^
SI	1				-	− 0.63^*^	0.88^*^
	2					0.18^*^	0.69^*^
	2.a					− 0.29^*^	0.78^*^
	3					− 0.37^*^	0.82^*^
	4					− 0.42^*^	0.82^*^
	5					0.06	0.70^*^
	Norm ^a^					− 0.50^*^	0.86^*^
PC	1					-	− 0.92^*^
	2						− 0.59^*^
	2.a						− 0.83^*^
	3						− 0.84^*^
	4						− 0.86^*^
	5						− 0.67^*^
	Norm ^a^						− 0.87^*^

^a^ Krampen [2]

^*^ *p* < .01

For hypothesis testing, exploratory structural equation models (ESEMs) [55, 56] were computed; both procedures using *R* [57]. This relatively novel approach is a theory-driven invariance test, which delivers fit indices that are compared across groups. It is especially useful when there are substantial cross-loadings between items given [55]. Two steps were carried out in this study. First, a configural invariance test was performed by assessing the fit of the hypothesized models across all the subsamples involved. This concerns the question of whether the model from the original I-SEE scale is replicable with the data at hand. Second, factor loadings and intercepts were forced to be equal across groups (metric and scalar invariance) [5, 58]. Model fit was again assessed by comparing the models with and without constraints applied. The assumption of equal factor loadings implies that scale intervals are the same across groups, which would allow, for example, covariance comparisons. If even intercepts are equal between groups, latent means can be directly compared between them [59]. As model fit indices, we report the root mean square error of approximation (RMSEA), standardized root mean square residual (SRMR), Tucker Lewis index and comparative fit index (TLI, CFI). Values of RMSEA and SRMR < 0.08 and CFI or TLI > 0.89 indicate an acceptable model fit [60, 61]. Following Chen’s [62] suggestions, the cutoff values for model fit differences are − 0.01 (∆CFI), 0.015 (∆RMSEA), and 0.03 (∆SRMR).

Explorative analyses at the level of items were conducted using IMINCE [63]. This procedure is based on unrestricted factor analysis and uses Bootstrap resampling to evaluate the significance of invariance tests when comparing two populations. From this framework, item loadings, indicators of discrepancy and of congruency are considered to allow for an examination of item and scale content.

## Results

### Explorative factor analyses

As an overview, the attempt to replicate the original four control facets resulted in a first factor comprising items relating to *Externality* (powerful others and chance orientation). The second factor consists of items representing *Internality* (self-concept of ability and internal control orientation). The theoretically intended structure could only be reproduced with large German samples. Factor solutions with two factors resemble each other across subsamples and can be easily interpreted.

For the transcultural total sample, the Kaiser‒Meyer‒Olkin measure of sampling adequacy was 0.904, and Bartlett’s test of Sphericity was significant, with *χ²* (406) = 10,966.06; *p* < .001. This resulted in three factors with eigenvalues > 1.3134 (Monte Carlo parallel analysis; CEFA 3.04 [64]. Altogether, they explained 45% of the item variance (for comparison, the manual reports 41% variance explained in the norm sample [2]). In accordance with the scree plot criterion [65, 66], three factors were rotated (direct oblimin rotation), clearly showing an *Internality* (factor 1) vs. *Externality* (factor 2) layout with only a few cross-loadings >|0.30|. The third factor remains inconclusive, assembling those items of the S-scale that are coded negatively and setting them into contrast with the P-and C-scales—most likely a method effect. The items’ communality values h² after extraction indicate how good the item loading can be predicted by the respective factor. Communalities range from low to very good, according to item loadings on a single factor. Compared with the corresponding corrected item‒total correlations, the overall moderate communalities are reasonable. Altogether, only the two secondary factors remain interpretable from the preliminary model when the transcultural total sample is considered: *Internality* and *Externality* (for all EFA details, see Table A2 in the Supplemental Materials).

Regarding subsamples, the parallel analysis criterion mostly resulted in two factors with explained variance shares between 34% and 40% (see Tables A3 to A7 in the Supplemental Materials). The larger German subsamples 2 and 2.a revealed three factors that explained up to 42% of the item variance. Since both samples very closely missed the criterion for the fourth factor, this solution was incorporated into the subsequent analyses. Concerning the items’ content within the factors, the subsamples each reveal differing features. As expected, the factor structures of the German samples assembled the S, I, P, and C items, thereby reproducing the four primary factors of Krampen’s scale.

The Polish structure is the only one that showed an inverted P/C factor, expressing not aligning with the content of the factor. The internality factor remains unaffected, raising the question of whether this indicates the presence of a second “internality-like” factor. Anyway, within the Polish sample, two factors remain interpretable and assemble the items’ content as expected from the secondary scales of the preliminary model: *Internality* and *Externality*.

The explorative factor analysis from the Kenyan sample in turn showed an internality factor and two P/C factors. In other words, the clustering of internal and external content from the secondary scales of the model is represented again here. However, it is not possible to determine from the content of the items in the third factor whether this is another type of externality. In any case, the exploratory nature of these findings and the small sample sizes require that the results be interpreted with care.

**Fig. 2:**
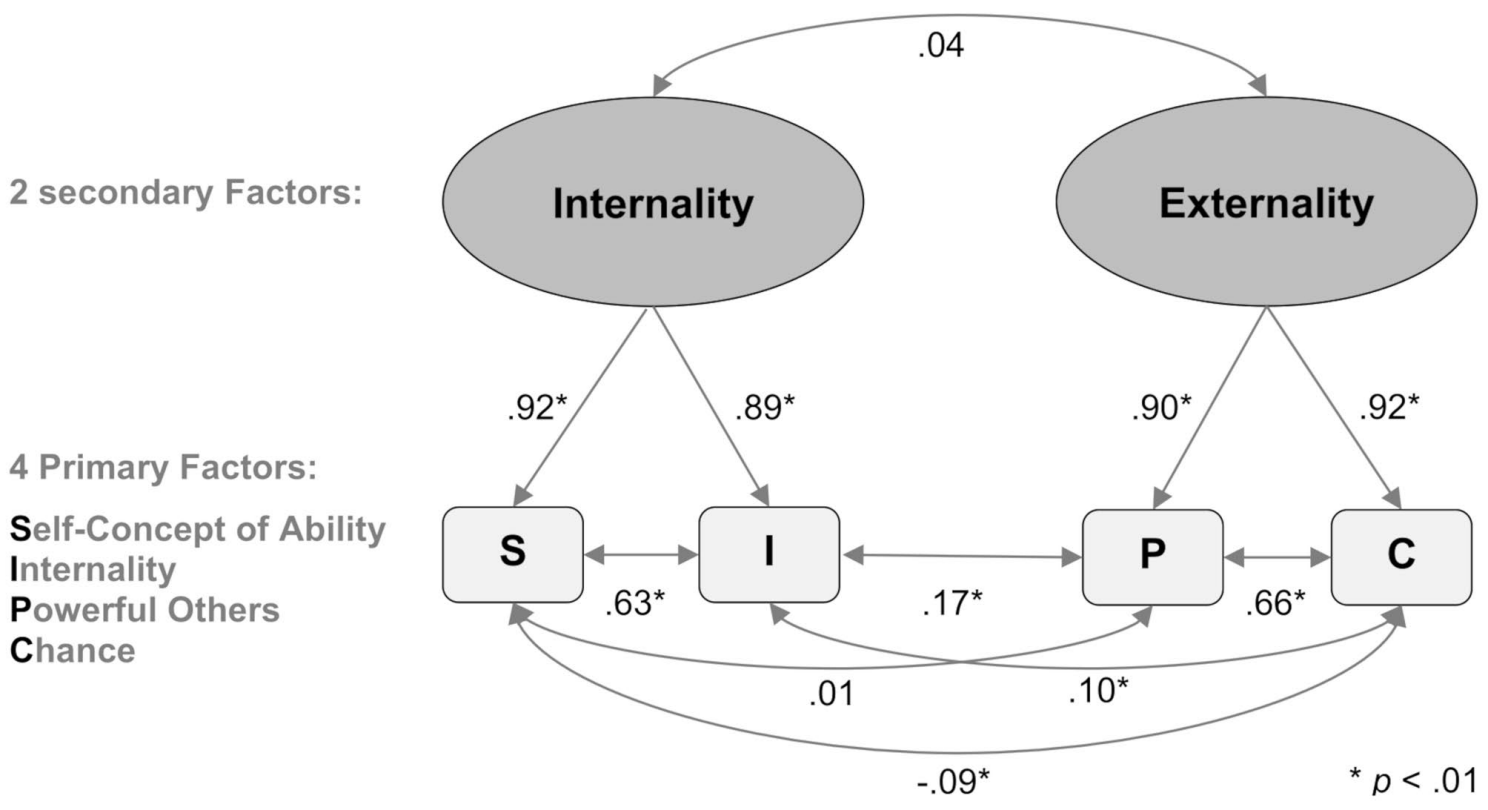
Preliminary model of the I-SEE structure (correlations from the total transcultural sample)

A comparison of the solutions between the subsamples revealed that the largest German subsample (2), which had four factors, was the closest to the results of the norm sample provided with the I-SEE scale. Reducing this sample to a representative German cluster (2.a) yields a merged solution in which the S items mix with the I and C-factors depending on how they are coded. A third factor seems to imply a P-factor at first glance, but upon closer inspection of the wording (e.g., repetition of the phrase “my life”), it is explained by a method effect. Additionally, only two factors remain interpretable in each subsample: *Internality* and *Externality* (see Fig. 2).

### Exploratory structural equation models

To evaluate the cross-cultural applicability of the four-factor model based on theory and the two-factor model based on observation, both models were included in a configural invariance test using exploratory structural equation modeling (ESEM). ESEM presupposes no single structure in which items load on only one factor [56].

The goodness‒of‒fit indices of the two- and four-factor solutions are shown in Table 4, and the models are visualized in Fig. 2. At first glance, the four-factor solution appears to fit the overall sample better than the two-factor solution, although both are barely acceptable. However, a closer look at the structural equations with item loadings (see Table A8 of the Supplementals) reveals that only two latent variables are interpretable. With two or four factors, the items cluster into two factors: an *Internality* factor (self-concept of ability and internal control orientation) and an *Externality* factor (powerful others and chance orientation). Restricting the model such that the item loadings and intercepts are equal between the subsamples results in considerably less goodness of fit [62, 66]. Consequently, scalar invariance is rejected.

**Table 4:** Goodness-of-fit indices of the two- and four-factorial I-SEE models (ESEM invariance test)

Model	Level			CFI	TLI	RMSEA	SRMR	Decision
2 Factors								
	configural	Total sample		0.725	0.724	0.068	0.066	
	scalar	Equal loadings and intercepts		0.576	0.592	0.086	0.095	
			∆	− 0.149	− 0.132	0.018	0.029	Reject
4 Factors								
	configural	Total sample		0.861	0.858	0.049	0.047	
	scalar	Equal loadings and intercepts		0.726	0.732	0.070	0.077	
			∆	− 0.135	− 0.126	0.021	0.03	Reject

Note. *CFI* = comparative fit index; *TLI* = tucker-Lewis Index; *RMSEA* = root mean square error of approximation; *SRMR* = standardized root mean square residual

### Analyses at item level

In order to assess cross-cultural similarities and differences regarding item content, we examined significant differences in item congruency and discrepancy using IMINCE [63] (for detailed results see the Supplements). Assuming a two-factor structure, the German representative sample (2.a) was set as target for comparisons with the Kenyan and Polish samples. Items were evaluated in a content analysis if they significantly differed from the expected values with regard to congruency and discrepancy or if they remained until the factors fit sufficiently well. Items that already showed a lack of reliability in the German sample were excluded (5, 16, 17, and 19; see Supplementals).

To state it directly: Of all the sample comparisons, only item 28 can be considered invariant. Perceiving oneself able to think about many alternatives to deal with challenging situations seems to make for some shared core of the construct.

When comparing German and Kenyan responses, several items stood out in terms of congruency or discrepancy. Within the Self-Concept of Ability scale (S), four of the seven items included differed significantly from expected values (items 8, 12, 16, and 20). However, only four items remained after the factors sufficiently correspond (4, 24, 28, and 32). The ratio of negatively coded items is always half, so a pure method effect can be ruled out. For the internality scale, only one item (27) was considered invariant in the sample comparison. This item states that “hard work” is the cause of achieving one’s intentions. Four items were found to be non-invariant (6, 11, 23, and 25), but no recognizable pattern emerged with regard to content. Two items from the powerful others scale (26 and 29) and two items from the chance scale were considered invariant. Again, two items from each scale were not considered invariant. Once again, a pattern of item content that leads to invariance or non-invariance seems hardly recognizable.

Comparing the German target with the Polish sample yields roughly the same number of invariant items as the Kenyan sample. However, only three items are found in both comparisons (28, 31, and 32). This comparison identified eight additional invariant items (2, 8, 10, 12, 13, 14, 18, and 25), while ten items indicated non-invariance. As with the previous comparison, no clear pattern emerged when comparing item content.

Regarding factor loadings, there were few significant differences between the samples compared. However, several items stood out with unexpected substantial cross-loadings (e.g., positive loadings on each factor instead of reversed signs) that were not shared by the target. In the Kenyan sample, one P-item and two C-items fell into this category (7, 10, and 13). In the Polish sample, item 23 loaded on both factors and referred to self-determination in life. A content analysis could not detect a common characteristic or a cause for the cross-loadings.

## Discussion

The I-SEE provides the capacity to assess control beliefs in relation to the sociocultural context, namely social, fatalistic, and spiritual or religious control beliefs. Although these aspects exhibit the most intercultural variance, they are equally important and at the same time, they are more challenging to operationalize. Although results indicate a common core of competence and control beliefs in the three cultural regions involved, the structure of the measure still differs significantly between the samples.

### A common core of competence and control beliefs

Two factors emerged consistently in the overall sample, as well as in each subsample. These factors properly represent the content of *internal* and *external* control beliefs, as expected from the theoretical background and as outlined by the original measure’s norm sample.

What seems reminiscent of historical approaches to control localization [9] is, in fact, more complex with respect to control orientations. All items from the four independent scales are combined into two factors that represent the theoretically expected secondary scales and their intercorrelations. Since we currently lack dependable data on the reliability and validity of this “W.E.I.R.D.” measure [67] in transcultural applications, these exploratory results encourage revising the theory and measure for transcultural use. Even with the small convenience samples from Kenya and Poland, which can in no way be regarded as representative, two independent control belief factors were found, which may help to inform future research [68, 69]. Nevertheless, there is serious doubt about transcultural comparability at the item and primary scale levels.

Interestingly, the German representative sample—which serves as a proxy for today’s German culture—did not successfully reproduce all four facets of control. Unlike the instrument’s tad old norm sample, the representative sample aligns more closely with the foreign samples. Whether this is a consequence of cultural changes in Germany or a result of sampling methods still needs to be examined.

### A measure not (yet) transferable across cultures

The theoretically proposed measurement model of four competence and control belief factors was not replicated as expected with the samples in this study. Two control factors remain interpretable with regard to content: *Internality* and *Externality*.

At first glance, the factor structures of the subsamples seem to resemble each other quite well. Factor loadings, correlational patterns, and item characteristics are all scattered by an expected value. However, when loadings and intercepts are forced to be equal across groups, the model’s fit decreases significantly. Considering that the Kenyan and Polish samples are neither large nor representative enough to provide solid evidence of transcultural (non-)invariance, the I-SEE measure is not ready for transcultural use in its current state. For example, why are there two externality factors detectable in Kenya? What does not aligning with the expected externality factor in the Polish sample mean? A notable detail is the number of items loading on both factors (even though they are assumed to be independent or even opposite of each other). Additionally, these items are not identical across samples. It would be premature to attribute these findings directly to culture-specific differences at this stage of research, especially with regard to meager sampling and the absence of a clear pattern in item content.

Nevertheless, attention is drawn again to those aspects of control relating to the impact of others, chance and religiosity. This contrasts with the long‒established unidimensional classic theories [9, 10]. It appears that there is more than one meaning for the same content, which allows an external item to shift to the internal side and vice versa. This phenomenon, which has already been observed in Western cultures [70], points to certain long under-noticed aspects of self-construal. Self-perception and the formation of control beliefs can be extended by social and spiritual or religious resources, and even by technical aids [71, 72]. The role of religiosity and spirituality within the context of control beliefs is still largely unresolved, but there are indications that they can produce internal as well as external beliefs, regardless of a particular religion. A distinct self-construal develops within socio-cultural contexts and should account for different interpretations when asked about one’s own internality and externality. Collectivist backgrounds can promote the activation of interdependent selves with powerful others perceived as internal resources [73]. In the case of chance beliefs, it is well documented that individuals and groups can adopt the internal belief that they can control the uncontrollable, even through a proxy relationship [74]. In summary, the exploration of culture-specific aspects of the construct of control is in its early stages.

The theoretical basis of the FKK/I-SEE scale [1, 2] provides four independent factors that sum to higher-level scales. Previous investigations using an emic-etic approach predicted that German, Kenyan, and Polish samples would exhibit psychological control differently, reflecting culture-specific features such as collectivism, interpersonal relations and religiosity. Consequently, a preliminary test for measurement invariance using pilot samples failed to an effectual extent. Concurrently, analyses of construct validity indicated, that a two-factor structure could prevail across the involved cultures. However, the construct content being measured requires further clarification.

### Limitations

This study provides insight into the challenges of transferring the construct of control beliefs transculturally. It is important to consider the limitations of this research. First, the convenience samples used for comparison with the German sample are relatively small and therefore have limited statistical power. Second, these samples lack representativeness, making them unsuitable for studying cultural populations, especially in a highly diverse country like Kenya. In Poland, the proportion of practicing believers is declining and has almost halved in the last 30 years [49]. Much has changed socially since before the COVID‒19 pandemic. More effort must be put into population-representative sampling, while considering the cultural contexts that may vary within a society and over time. Then, a contemporary model of competence and control beliefs built on Krampen’s action theoretical model [17] would have to be embedded in these contexts. This model would also have to consider related personality and external variables (e.g., values, social skills, and social axioms). The predictive value must be evaluated using objective or behavioral criteria (e.g., with regard to well-being, job performance, and learning motivation). Most importantly, however, we must pursue the fruitful emic-etic approach to the problem of capturing universal and culture-specific aspects with a reliable instrument [69]. This study only provides a brief snapshot at the beginning of a long-term evaluation and adaptation process. More comparable and complementary research is needed to estimate the true significance of the results, linking them to earlier reviews of related constructs [31, 36].

## Conclusion

Our global society is networked—not only digitally and in terms of information, but also with regard to the flow of money, goods, services, and many other resources. Migration and cultural diversity are now part of everyday life. An increasing demand for diagnostics in education, healthcare, workplaces, and other areas has led to a greater focus on the efficiency and universality of psychological instruments, including those aimed at control beliefs [35]. In the context of transcultural applications, severely curtailing culturally specific content to leave only a universal core for assessment can lead to poor results [77]. The importance of universal yet efficient instruments is still indisputable [24].

In this study, a well-established and widely-accepted German personality test for competence and control beliefs [1, 2] was used across time and borders, from nearby to more distant cultural regions. Details were provided on the factorial validity and measurement invariance examined, showing the I-SEE measure’s great potential—but as well the necessity—for adaptation to today’s transcultural personality psychology. Two factors of internal and external control beliefs seem to hold for a common core of the construct when applied in different cultural regions and their content is basically represented by the two secondary scales of the I-SEE. At the same time, the four primary factors could not be replicated and it is evident that there are reasonable doubts not only about measurement invariance, but also about construct validity. The content of items aimed at competence and control beliefs needs to be revised in the transcultural context, including not only social and fatalistic aspects (e.g., social axioms and social skills in Germany and Kenya) [75, 76], but also the questions of whether and when such content can be perceived as either internal or external. What has previously been considered an external source of control can just as easily be perceived as an internal resource (e.g., a wealthy relative, a clever colleague, an appeased deity or fate, or even a technical aid) [71, 72]. A preliminary step has been taken toward updating for the German region [35].

The next step is to evaluate the role of competence and control beliefs as self-related constructs [11], which serve both independent and interdependent functions [73]. Recent approaches, such as that of Fan et al. [78] and findings from social psychology [79], have paved the way for including interdependence and relatedness aspects in a transcultural personality model. It is not recommended to repeatedly analyze factor structures using the same items. Instead, a revision starting at the basis of content validity is required. This involves critically examining the statements used as test items and formulating new items that allow for internal content from the socio-cultural embedding. Furthermore, this requires examining the specific manner in which items fulfill independent and interdependent functions within a culturally specific context.

## Supplementary Information

Below is the link to the electronic supplementary material.

Supplementary Material 1

## Data Availability

The datasets used and analyzed during this study are available from the corresponding author upon request.

## Footnotes

1 For better readability and to avoid confusion, we are going to use the term ‘internal’ rather than Krampen’s Self-efficacy.

## Abbreviations

C: Chance
CFI: Comparative fit index
COVID-19: Coronavirus disease 2019
EFA: Exploratory factor analysis
ESEM: Exploratory structural equation modeling
FKK: Fragebogen zu Kompetenz- und Kontrollüberzeugungen [2]
I-SEE: Inventory for Self-Efficacy and Externality [1]
I: Internality
P: Powerful others
PC: Powerful others and chance; externality
RMSEA: Root mean square error of approximation
S: Self-concept of ability
SI: Self-concept of ability and internality; self-efficacy
SIPC: Generalized control orientation; self-efficacy adjusted for externality
SRMR: Standardized root mean square residual
TLI: Tucker Lewis index
W.E.I.R.D.: Western, educated, industrial, rich, democratic [67]
