# Supplementary material for: Competence and control beliefs in three cultures: a German personality test applied at home, in Kenya and Poland
Source: BMC Psychol. 2025 Sep 10;13:1008. doi: 10.1186/s40359-025-03416-2 (PMC12421771; doi:10.1186/s40359-025-03416-2)
Supplement: Supplementary file 1 — Supplementary Material 1 [file 40359_2025_3416_MOESM1_ESM.pdf]

**Table A1**

*Means (M), Standard Deviations (SD) and Corrected Item-Total-Correlations (CITC) of I-SEE Items*

| Item | Scale | *M* | *SD* | *CITC* | *M* | *SD* | *CITC* | *M* | *SD* | *CITC* |
|---|---|---|---|---|---|---|---|---|---|---|
| | | Norm sample[a] | | | Total sample 5 | | | German sample 1 | | |
| 1 | I | 4.0 | 1.3 | .36 | 4.1 | 1.1 | .20 | 4.0 | 1.1 | .37 |
| 2 | C | 3.3 | 1.3 | .44 | 3.3 | 1.4 | .38 | 3.3 | 1.1 | .40 |
| 3 | P | 3.3 | 1.3 | .50 | 3.3 | 1.4 | .43 | 3.6 | 1.2 | .63 |
| 4 | S | 4.5 | 1.3 | .50 | 3.9 | 1.6 | .36 | 4.5 | 1.2 | .50 |
| 5 | I | 3.3 | 1.4 | .33 | 3.0 | 1.5 | .09 | 2.7 | 1.1 | .16 |
| 6 | I | 3.9 | 1.2 | .50 | 4.1 | 1.3 | .39 | 4.1 | 1.1 | .46 |
| 7 | C | 3.2 | 1.3 | .45 | 2.9 | 1.4 | .40 | 3.0 | 1.3 | .70 |
| 8 | S | 3.4 | 1.5 | .37 | 3.5 | 1.6 | .25 | 3.5 | 1.2 | .39 |
| 9 | C | 3.8 | 1.2 | .39 | 3.5 | 1.3 | .43 | 3.7 | 1.1 | .52 |
| 10 | P | 2.9 | 1.3 | .45 | 2.7 | 1.3 | .34 | 2.6 | 1.1 | .38 |
| 11 | I | 4.1 | 1.2 | .36 | 4.4 | 1.2 | .33 | 4.2 | 1.2 | .18 |
| 12 | S | 4.0 | 1.3 | .44 | 4.0 | 1.4 | .51 | 4.4 | 1.2 | .49 |
| 13 | C | 3.4 | 1.2 | .56 | 2.9 | 1.3 | .51 | 3.1 | 1.2 | .76 |
| 14 | P | 3.4 | 1.3 | .54 | 3.2 | 1.3 | .45 | 3.2 | 1.1 | .51 |
| 15 | C | 3.4 | 1.3 | .44 | 2.9 | 1.5 | .46 | 3.3 | 1.3 | .60 |
| 16 | S | 4.1 | 1.2 | .33 | 4.5 | 1.4 | .39 | 4.5 | 1.2 | .14 |
| 17 | P | 2.9 | 1.3 | .36 | 2.6 | 1.6 | .37 | 2.4 | 1.2 | .41 |
| 18 | C | 3.2 | 1.4 | .51 | 2.8 | 1.6 | .52 | 3.1 | 1.5 | .51 |
| 19 | P | 3.6 | 1.3 | .34 | 4.1 | 1.4 | .42 | 4.4 | 1.0 | .24 |
| 20 | S | 3.8 | 1.2 | .56 | 3.7 | 1.4 | .56 | 3.5 | 1.1 | .54 |
| 21 | C | 2.8 | 1.2 | .41 | 2.5 | 1.4 | .46 | 2.5 | 1.1 | .30 |
| 22 | P | 3.2 | 1.3 | .50 | 3.3 | 1.6 | .61 | 3.6 | 1.3 | .45 |
| 23 | I | 4.4 | 1.1 | .53 | 4.7 | 1.4 | .53 | 4.7 | 1.1 | .61 |
| 24 | S | 4.0 | 1.3 | .58 | 3.5 | 1.6 | .64 | 3.6 | 1.3 | .64 |
| 25 | I | 4.4 | 1.1 | .47 | 4.5 | 1.4 | .58 | 4.6 | 1.0 | .54 |
| 26 | P | 3.5 | 1.2 | .29 | 3.1 | 1.5 | .49 | 3.3 | 0.9 | .09 |
| 27 | I | 4.4 | 1.1 | .47 | 4.4 | 1.5 | .56 | 4.6 | 0.9 | .32 |
| 28 | S | 4.1 | 1.1 | .59 | 4.2 | 1.4 | .63 | 4.2 | 1.0 | .58 |
| 29 | P | 3.3 | 1.2 | .43 | 3.4 | 1.6 | .48 | 3.2 | 1.2 | .32 |
| 30 | I | 3.8 | 1.2 | .40 | 4.4 | 1.5 | .55 | 3.7 | 1.2 | .43 |
| 31 | C | 3.7 | 1.4 | .39 | 2.9 | 1.6 | .49 | 3.0 | 1.4 | .45 |
| 32 | S | 4.1 | 1.1 | .60 | 3.9 | 1.6 | .65 | 4.2 | 1.0 | .54 |

| Item | Scale | *M* | *SD* | *CITC* | *M* | *SD* | *CITC* | *M* | *SD* | *CITC* | *M* | *SD* | *CITC* |
|---|---|---|---|---|---|---|---|---|---|---|---|---|---|
| | | German sample 2 | | | German sample 2.a | | | Kenyan sample 3 | | | Polish sample 4 | | |
| 1 | I | 4.1 | 1.0 | .20 | 4.1 | 1.0 | .30 | 4.4 | 1.5 | .13 | 4.2 | 1.1 | .13 |
| 2 | C | 3.5 | 1.4 | .34 | 3.6 | 1.2 | .44 | 2.3 | 1.5 | .47 | 3.2 | 1.3 | .55 |
| 3 | P | 3.4 | 1.3 | .39 | 3.4 | 1.3 | .48 | 2.5 | 1.5 | .48 | 3.2 | 1.4 | .51 |
| 4 | S | 4.0 | 1.6 | .35 | 4.2 | 1.4 | .51 | 3.8 | 1.4 | .43 | 3.0 | 1.5 | .36 |
| 5 | I | 3.0 | 1.5 | .07 | 3.3 | 1.4 | .26 | 3.3 | 1.8 | .12 | 3.1 | 1.5 | .18 |
| 6 | I | 4.1 | 1.3 | .38 | 4.2 | 1.0 | .48 | 4.6 | 1.3 | .34 | 4.1 | 1.1 | .48 |
| 7 | C | 2.8 | 1.3 | .38 | 2.9 | 1.3 | .39 | 2.9 | 1.6 | .24 | 3.8 | 1.4 | .46 |
| 8 | S | 3.6 | 1.6 | .26 | 3.6 | 1.4 | .39 | 3.4 | 1.6 | -.13 | 2.9 | 1.5 | .41 |
| 9 | C | 3.6 | 1.2 | .41 | 3.6 | 1.2 | .52 | 1.9 | 1.1 | .43 | 4.0 | 1.4 | .51 |
| 10 | P | 2.6 | 1.2 | .32 | 2.8 | 1.2 | .38 | 2.4 | 1.5 | .38 | 3.1 | 1.4 | .51 |
| 11 | I | 4.4 | 1.2 | .34 | 4.2 | 1.0 | .38 | 4.6 | 1.4 | .35 | 4.2 | 1.4 | .33 |
| 12 | S | 4.0 | 1.4 | .55 | 4.1 | 1.3 | .51 | 4.6 | 1.6 | .25 | 3.9 | 1.5 | .46 |
| 13 | C | 3.0 | 1.3 | .56 | 3.0 | 1.2 | .56 | 2.1 | 1.3 | .55 | 2.9 | 1.4 | .63 |
| 14 | P | 3.4 | 1.2 | .42 | 3.4 | 1.3 | .44 | 1.9 | 1.2 | .55 | 2.9 | 1.2 | .52 |
| 15 | C | 2.9 | 1.6 | .47 | 3.0 | 1.2 | .51 | 2.3 | 1.5 | .40 | 3.2 | 1.4 | .23 |
| 16 | S | 4.5 | 1.5 | .42 | 4.5 | 1.1 | .16 | 4.8 | 1.3 | .12 | 4.3 | 1.2 | .26 |
| 17 | P | 2.5 | 1.7 | .43 | 2.7 | 1.1 | .22 | 2.8 | 1.5 | .12 | 3.1 | 1.2 | .39 |
| 18 | C | 2.8 | 1.7 | .52 | 2.8 | 1.4 | .52 | 2.3 | 1.5 | .38 | 3.3 | 1.5 | .46 |
| 19 | P | 4.3 | 1.4 | .41 | 4.1 | 1.2 | .23 | 2.5 | 1.5 | .31 | 4.1 | 1.3 | .24 |
| 20 | S | 3.8 | 1.4 | .57 | 4.1 | 1.1 | .44 | 3.6 | 1.4 | .14 | 3.8 | 1.2 | .61 |
| 21 | C | 2.3 | 1.4 | .52 | 2.5 | 1.1 | .58 | 2.8 | 1.4 | .39 | 3.4 | 1.2 | .28 |
| 22 | P | 3.3 | 1.6 | .62 | 3.2 | 1.2 | .44 | 2.3 | 1.5 | .36 | 3.2 | 1.4 | .67 |
| 23 | I | 4.7 | 1.4 | .64 | 4.9 | 0.8 | .47 | 3.7 | 1.8 | .19 | 5.1 | 0.9 | .39 |
| 24 | S | 3.7 | 1.7 | .69 | 3.9 | 1.3 | .57 | 3.3 | 1.6 | .26 | 2.9 | 1.5 | .55 |
| 25 | I | 4.5 | 1.4 | .60 | 4.6 | .9 | .48 | 4.4 | 1.2 | .41 | 4.7 | 1.0 | .55 |
| 26 | P | 3.1 | 1.5 | .50 | 3.2 | 1.1 | .32 | 2.7 | 1.4 | .39 | 3.3 | 1.3 | .46 |
| 27 | I | 4.3 | 1.6 | .38 | 4.5 | 1.0 | .38 | 5.0 | 1.3 | .51 | 4.7 | 0.9 | .44 |
| 28 | S | 4.1 | 1.5 | .68 | 4.3 | 1.0 | .56 | 4.5 | 1.3 | .39 | 4.5 | 1.0 | .51 |
| 29 | P | 3.3 | 1.6 | .55 | 3.4 | 1.0 | .35 | 2.9 | 1.5 | .41 | 4.4 | 1.1 | -,03 |
| 30 | I | 3.7 | 1.6 | .56 | 3.9 | 1.2 | .49 | 4.9 | 1.1 | .52 | 4.9 | 0.9 | .50 |
| 31 | C | 2.8 | 1.6 | .50 | 3.1 | 1.3 | .45 | 2.6 | 1.6 | .36 | 3.7 | 1.5 | .32 |
| 32 | S | 4.3 | 1.4 | .72 | 4.5 | 0.9 | .54 | 5.0 | 1.1 | .33 | 4.5 | 1.1 | .40 |

[a]*Krampen (1991)*

**Table A2**

*Results From Exploratory Factor Analysis of the I-SEE Questionnaire*

| Extraction criterion | Parallel analysis (Eigenvalue > 1.3134) | | | | and Scree plot | | | |
|---|---|---|---|---|---|---|---|---|
| Loading on factor | 1 | 2 | | | 1 | 2 | 3 | |
| Explained variance % | 22 | 17 | | | 22 | 17 | 5 | |
| | | | $h^2$ | | | | | $h^2$ |
| 28 - S | **.82** | | .67 | 25 - I | **.83** | | | .67 |
| 25 - I | **.81** | | .66 | 28 - S | **.82** | | | .67 |
| 32 - S | **.78** | | .61 | 32 - S | **.77** | | | .61 |
| 27 - I | **.69** | | .48 | 24 - S (R) | **.70** | .11 | **.34** | .49 |
| 23 - I | **.67** | | .45 | 27 - I | **.67** | | -.15 | .45 |
| 24 – S (R) | **.64** | -.16 | .43 | 23 - I | **.67** | | | .52 |
| 20 - S | **.61** | | .37 | 20 - S | **.63** | | | .38 |
| 30 - I | **.58** | | .34 | 16 - S | **.54** | | | .43 |
| 16 - S | **.55** | | .31 | 30 - I | **.53** | -.26 | **-.32** | .31 |
| 6 - I | **.41** | -.24 | .22 | 12 - S (R) | **.48** | -.23 | **.38** | .22 |
| 11 - I | **.32** | | .10 | 26 - P | **.45** | **.30** | -.21 | .11 |
| 13 - C | -.20 | **.60** | .39 | 6 - I | **.41** | -.16 | .14 | .42 |
| 18 - C | .16 | **.59** | .39 | 29 - P | **.39** | .21 | -.27 | .40 |
| 22 - P | .22 | **.59** | .41 | 19 - P | **.35** | **.33** | | .42 |
| 3 - P | -.16 | **.55** | .32 | 11 - I | **.30** | -.13 | | .44 |
| 14 - P | -.16 | **.54** | .31 | 3 - P | | **.67** | | .43 |
| 21 - C | .25 | **.54** | .36 | 14 - P | | **.66** | | .46 |
| 10 - P | | **.51** | .27 | 2 - C | | **.59** | | .27 |
| 7 - C | -.14 | **.51** | .29 | 13 - C | -.15 | **.56** | -.14 | .30 |
| 12 - S (R) | **.46** | **-.50** | .45 | 22 - P | .25 | **.45** | -.23 | .46 |
| 9 - C | | **.49** | .24 | 9 - C | | **.45** | -.11 | .26 |
| 31 - C | **.31** | **.47** | .33 | 10 - P | | **.32** | -.29 | .33 |
| 15 - C | .22 | **.46** | .26 | 15 - C | .23 | .28 | -.25 | .26 |
| 26 - P | **.43** | **.45** | .41 | 21 - C | .21 | | **-.58** | .40 |
| 2 - C | | **.44** | .20 | 4 - S (R) | **.32** | | **.56** | .31 |
| 17 - P | .21 | **.42** | .23 | 17 - P | .17 | | **-.52** | .32 |
| 29 - P | **.39** | **.41** | .33 | 18 - C | .15 | .27 | **-.44** | .33 |
| 4 - S (R) | .26 | **-.40** | .22 | 7 - C | -.16 | .19 | **-.43** | .35 |
| 19 - P | **.32** | **.37** | .25 | 31 - C | **.31** | .21 | **-.33** | .26 |

*Note*. $N$ = 1,084 (total sample no. 5). The extraction method was principal axis analysis with direct oblimin rotation. Loadings >|0.30| are in bold, loadings < |0.10| are omitted. Reverse scored items are denoted with an (R). Items No. 1, 5, and 8 were left out due to lack of reliability. Items from Krampen (1991).

**Table A3**

*Results From Exploratory Factor Analysis of the I-SEE Questionnaire*

| Extraction criterion | Parallel analysis (Eigenvalue > 2.3346) | | | Scree plot | | | | |
|---|---|---|---|---|---|---|---|---|
| Loading on factor | 1 | 2 | | | 1 | 2 | 3 | |
| Explained variance % | 28 | 11 | | | 28 | 11 | 9 | |
| | | | h² | | | | | h² |
| 13 - C | **.80** | | .69 | 7 - C | **.83** | | | .68 |
| 7 - C | **.72** | | .58 | 13 - C | **.80** | | | .70 |
| 18 - C | **.71** | .20 | .44 | 24 - S (R) | **-.60** | .24 | | .52 |
| 23 - I | **-.70** | | .49 | 9 - C | **.59** | | | .39 |
| 15 - C | **.59** | | .35 | 15 - C | **.59** | | | .36 |
| 9 - C | **.58** | | .38 | 18 - C | **.58** | .23 | -.27 | .45 |
| 31 - C | **.57** | .11 | .29 | 3 - P | **.55** | **-.33** | .20 | .50 |
| 24 - S (R) | **-.60** | .29 | .51 | 22 - P | **.53** | | | .28 |
| 2 - C | **.50** | .11 | .23 | 10 - P | **.52** | -.15 | .23 | .33 |
| 22 - P | **.50** | | .27 | 31 - C | **.50** | .13 | -.15 | .28 |
| 27 - I | **-.50** | | .19 | 2 - C | **.50** | .13 | | .23 |
| 6 - I | **-.40** | .23 | .27 | 14 - P | **.41** | **-.34** | .28 | .38 |
| 3 - P | **.38** | **-.36** | .37 | 6 - I | **-.34** | .20 | .20 | .28 |
| 10 - P | **.36** | -.18 | .21 | 8 - S (R) | **-.34** | .27 | | .23 |
| 20 - S | **-.40** | **.33** | .33 | 4 - S (R) | .15 | **.79** | | .58 |
| 29 - P | **.32** | | .11 | 32 - S | .16 | **.73** | .10 | .51 |
| 4 -S (R) | **.30** | **.81** | .57 | 17 - P | | **-.65** | -.25 | .57 |
| 32 - S | .21 | **.76** | .51 | 28 - S | | **.63** | .12 | .42 |
| 17 - P | .11 | **-.68** | .55 | 12 - S (R) | -.20 | **.62** | -.12 | .49 |
| 28 - S | | **.67** | .42 | 21 - C | .18 | **-.57** | -.12 | .45 |
| 12 - S (R) | | **.64** | .44 | 30 - I | -.20 | .30 | .21 | .24 |
| 21 - C | .14 | **-.61** | .46 | 1 - I | .24 | .14 | **.66** | .44 |
| 14 - P | .22 | **-.36** | .23 | 23 - I | **-.46** | | **.61** | .71 |
| 25 - I | -.20 | **.35** | .24 | 20 - S | -.19 | .30 | **.52** | .42 |
| 30 - I | -.20 | **.32** | .22 | 25 - I | | **.34** | **.50** | .24 |
| 8 -S (R) | -.20 | .29 | .19 | 27 - I | -.25 | -.10 | **.49** | .34 |
| 1 - I | | .13 | .03 | 29 - P | .22 | | -.26 | .14 |

*Note*. $N$ = 52 (German sample no. 1.) The extraction method was principal axis analysis with direct oblimin rotation. Loadings >|0.30| are in bold, loadings < |0.10| are omitted. Reverse scored items are denoted with an (R). Items No. 5, 11, 16, 19, and 26 were left out due to lack of reliability. Items from Krampen (1991).

**Table A4**

*Results From Exploratory Factor Analysis of the I-SEE Questionnaire*

| Extraction criterion | Parallel analysis (Eigenvalue > 1.3349) | | | | | Scree plot | | | | |
|---|---|---|---|---|---|---|---|---|---|---|
| Loading on factor | 1 | 2 | 3 | | | 1 | 2 | 3 | 4 | |
| Explained variance % | 25 | 11 | 6 | | | 25 | 11 | 6 | 5 | |
| | | | | h² | | | | | | h² |
| 13 - C | **-.66** | | | .42 | 24 - S (R) | **.70** | | -.11 | | .51 |
| 2 - C | **-.62** | .19 | | .35 | 12 - S (R) | **.57** | | -.17 | -.22 | .57 |
| 3 - P | **-.59** | | **.32** | .45 | 4 - S (R) | **.56** | | -.17 | | .39 |
| 9 - C | **-.57** | | -.18 | .34 | 32 - S | **.55** | **.40** | .15 | | .57 |
| 14 - P | **-.54** | | **.33** | .40 | 28 - S | **.52** | **.42** | .17 | | .55 |
| 10 - P | **-.53** | | | .29 | 8 - S (R) | **.50** | | | | .30 |
| 18 - C | **-.49** | -.17 | | .32 | 20 - S | **.41** | **.35** | .14 | | .36 |
| 21 - C | **-.48** | -.19 | | .31 | 30 - I | | **.59** | -.27 | .16 | .40 |
| 15 - C | **-.47** | | -.13 | .25 | 23 - I | | **.52** | -.21 | -.12 | .44 |
| 7 - C | **-.46** | -.19 | | .31 | 27 - I | | **.50** | -.13 | | .28 |
| 22 - P | **-.43** | | .15 | .25 | 6 - I | .22 | **.46** | | | .37 |
| 31 - C | **-.39** | | -.11 | .16 | 25 - I | **.36** | **.42** | | | .45 |
| 29 - P | -.29 | | .20 | .13 | 11 - I | | **.37** | | -.10 | .14 |
| 32 - S | | **.75** | | .57 | 3 - P | | | **.65** | | .51 |
| 28 - S | -.13 | **.74** | -.12 | .56 | 14 - P | | -.11 | **.62** | | .45 |
| 20 - S | -.12 | **.59** | -.10 | .36 | 10 - P | -.13 | | **.41** | .18 | .31 |
| 24 - S (R) | .20 | **.58** | | .42 | 22 - P | -.17 | | **.41** | | .28 |
| 25 - I | | **.54** | -.20 | .44 | 29 - P | | | **.37** | | .15 |
| 12 -S (R) | **.45** | **.48** | | .55 | 31 - C | | | -.13 | **.61** | .31 |
| 4 - S (R) | .27 | **.45** | | .34 | 15 - C | | | | **.57** | .32 |
| 6 - I | | **.43** | -.29 | .36 | 13 - C | | | .24 | **.54** | .45 |
| 8 - S (R) | .26 | **.41** | .13 | .28 | 9 - C | | .13 | | **.51** | .35 |
| 30 - I | | .12 | **-.60** | .41 | 2 - C | .14 | | .25 | **.50** | .37 |
| 27 - I | | .20 | **-.42** | .27 | 18 - C | -.18 | | .12 | **.41** | .33 |
| 23 - I | .22 | **.30** | **-.41** | .43 | 21 - C | -.22 | | .11 | **.39** | .31 |
| 11 - I | | .17 | -.21 | .09 | 7 - C | -.23 | | .20 | .29 | .30 |

*Note. N* = 833 (German sample no. 2). The extraction method was principal axis analysis with direct oblimin rotation. Loadings >|0.30| are in bold, loadings < |0.10| are omitted. Reverse scored items are denoted with an (R). Items No. 1, 5, 16, 17, 19, and 26 were left out due to lack of reliability. Items from Krampen (1991).

**Table A5**

*Results From Exploratory Factor Analysis of the I-SEE Questionnaire*

| Extraction criterion | Parallel analysis (Eigenvalue > 1.7565) and Scree plot | | | | | | | | | |
|---|---|---|---|---|---|---|---|---|---|---|
| Loading on factor | 1 | 2 | 3 | | | 1 | 2 | 3 | 4 | |
| Explained variance % | 21 | 13 | 7 | | | 21 | 13 | 7 | 5 | |
| | | | | h² | | | | | | h² |
| 12 - S (R) | **-.65** | .21 | | .54 | 24 - S (R) | **-.67** | | | | .54 |
| 9 - C | **.65** | .28 | | .44 | 4 - S (R) | **-.53** | .13 | | | .38 |
| 21 - C | **.62** | | .11 | .44 | 12 - S (R) | **-.53** | | -.20 | -.26 | .57 |
| 24 - S (R) | **-.59** | .29 | | .44 | 8 - S (R) | **-.51** | | -.14 | -.11 | .34 |
| 18 - C | **.57** | | .13 | .40 | 7 - C | **.30** | | | **.28** | .25 |
| 15 - C | **.54** | | | .33 | 25 - I | -.20 | **.57** | | -.17 | .48 |
| 8 - S (R) | **-.52** | .11 | | .29 | 23 - I | | **.56** | -.24 | -.10 | .43 |
| 7 - C | **.46** | -.13 | | .29 | 6 - I | | **.56** | | | .37 |
| 4 - S (R) | **-.46** | **.32** | | .33 | 30 - I | | **.53** | **-.34** | **.32** | .44 |
| 13 - C | **.43** | | .26 | .31 | 32 - S | **-.40** | **.50** | .20 | | .51 |
| 31 - C | **.39** | | .14 | .20 | 28 - S | **-.40** | **.50** | .18 | | .51 |
| 2 - C | **.35** | .24 | .27 | .28 | 20 - S | **-.31** | **.48** | .13 | .14 | .41 |
| 10 - P | **.32** | | **.30** | .24 | 27 - I | | **.47** | | | .23 |
| 28 - S | -.18 | **.65** | .25 | .51 | 11 - I | .14 | **.41** | | | .16 |
| 32 - S | -.21 | **.65** | .26 | .52 | 1 - I | -.20 | **.36** | .18 | | .22 |
| 20 - S | | **.61** | .19 | .41 | 3 - P | | | **.67** | | .49 |
| 25 - I | -.25 | **.58** | | .46 | 14 - P | | -.14 | **.57** | .11 | .42 |
| 30 - I | **.30** | **.57** | **-.33** | .44 | 10 - P | **.31** | .13 | **.45** | | .33 |
| 6 - I | | **.56** | -.12 | .35 | 22 - P | | | **.44** | | .24 |
| 23 - I | | **.53** | **-.30** | .41 | 29 - P | | | **.33** | | .11 |
| 27 - I | .15 | **.44** | - .12 | .21 | 26 - P | | | **.32** | | .15 |
| 1 - I | -.15 | **.41** | .17 | .21 | 13 - C | -.18 | -.20 | | **.72** | .51 |
| 11 - I | .11 | **.32** | | .11 | 9 - C | .21 | .25 | | **.56** | .45 |
| 3 - P | .14 | | **.62** | .46 | 15 - C | .12 | | | **.53** | .35 |
| 14 - P | .16 | -.12 | **.57** | .42 | 21 - C | .22 | | | **.52** | .44 |
| 22 - P | .16 | | **.40** | .22 | 2 - C | -.13 | | .16 | **.51** | .33 |
| 29 - P | | | **.33** | .11 | 31 - C | | | | **.48** | .25 |
| 26 - P | .19 | | .27 | .14 | 18 - C | .22 | -.11 | | **.47** | .41 |

*Note. N* = 206 (German sample no. 2.a). The extraction method was principal axis analysis with direct oblimin rotation. Loadings >|0.30| are in bold, loadings < |0.10| are omitted. Reverse scored items are denoted with an (R). Items No. 5, 16, 17, and 19 were left out due to lack of reliability. Items from Krampen (1991).

**Table A6**

*Results From Exploratory Factor Analysis of the I-SEE Questionnaire*

| Extraction criterion | Parallel analysis (Eigenvalue > 2.0475) | | | | Scree plot | | | |
|---|---|---|---|---|---|---|---|---|
| Loading on factor | 1 | 2 | | | 1 | 2 | 3 | |
| Explained variance % | 21 | 13 | | | 21 | 13 | 7 | |
| | | | $h^2$ | | | | | $h^2$ |
| 13 - C | **.66** | -.15 | .50 | 10 - P | **.67** | .16 | | .22 |
| 14 - P | **.63** | -.20 | .50 | 21 - C | **.54** | | | .31 |
| 9 - C | **.53** | | .30 | 3 - P | **.51** | -.13 | | .34 |
| 10 - P | **.50** | | .24 | 4 - S (R) | **-.45** | .22 | | .27 |
| 22 - P | **.49** | | .24 | 13 - C | **.44** | -.13 | **.37** | .49 |
| 21 - C | **.48** | | .23 | 22 - P | **.40** | | .21 | .25 |
| 18 - C | **.48** | | .24 | 18 - C | **.36** | | .23 | .24 |
| 29 - P | **.47** | .18 | .21 | 2 - C | .27 | -.23 | .25 | .27 |
| 3 - P | **.46** | -.18 | .28 | 32 - S | **.31** | **.79** | -.22 | .64 |
| 2 - C | **.42** | -.24 | .28 | 28 - S | .11 | **.77** | | .58 |
| 15 - C | **.41** | | .16 | 27 - I | -.10 | **.64** | .19 | .45 |
| 26 - P | **.39** | | .15 | 30 - I | | **.60** | | .35 |
| 31 - C | **.37** | .15 | .13 | 25 - I | | **.60** | .11 | .38 |
| 19 - P | **.35** | -.15 | .17 | 20 - S | **-.34** | **.40** | .18 | .31 |
| 28 - S | | **.77** | .57 | 11 - I | -.11 | **.39** | -.12 | .49 |
| 32 - S | | **.71** | .49 | 6 - I | | **.37** | | .16 |
| 27 - I | | **.67** | .43 | 29 - P | | .14 | **.59** | .35 |
| 25 - I | | **.62** | .38 | 9 - C | .12 | | **.53** | .36 |
| 30 - I | | **.60** | .36 | 19 - P | | -.20 | **.53** | .35 |
| 20 - S | -.11 | **.44** | .22 | 26 - P | | | **.47** | .22 |
| 11 - I | -.18 | **.40** | .22 | 15 - C | | | **.45** | .22 |
| 6 - I | | **.38** | .16 | 14 - P | **.33** | -.20 | **.45** | .49 |
| 4 - S (R) | -.26 | .27 | .17 | 31 - C | .12 | .13 | **.32** | .14 |

*Note*. *N* = 91 (Kenyan sample no. 3.) The extraction method was principal axis analysis with direct oblimin rotation. Loadings >|0.30| are in bold, loadings < |0.10| are omitted. Reverse scored items are denoted with an (R). Items No. 1, 5, 7, 8, 12, 16, 17, 23, and 24 were left out due to lack of reliability. Items from Krampen (1991).

**Table A7**

*Results From Exploratory Factor Analysis of the I-SEE Questionnaire*

| Extraction criterion | Parallel analysis (Eigenvalue > 1.9539) | | | | Scree plot | | | |
|---|---|---|---|---|---|---|---|---|
| Loading on factor | 1 | 2 | | | 1 | 2 | 3 | |
| Explained variance % | 26 | 14 | | | 26 | 14 | 7 | |
| | | | h² | | | | | h² |
| 13 - C | **-.75** | -.13 | .62 | 10 - P | **-.73** | | | .54 |
| 10 - P | **-.73** | .10 | .50 | 13 - C | **-.67** | -.14 | -.19 | .62 |
| 7 - C | **-.69** | .12 | .45 | 22 - P | **-.67** | -.10 | | .44 |
| 22 - P | **-.64** | | .43 | 2 - C | **-.61** | | | .48 |
| 3 - P | **-.62** | -.21 | .48 | 3 - P | **-.58** | -.22 | -.11 | .49 |
| 4 - S (R) | **.57** | | .32 | 7 - C | **-.58** | .11 | -.23 | .44 |
| 18 - C | **-.56** | | .33 | 26 - P | **-.52** | | | .27 |
| 2 - C | **-.55** | | .30 | 31 - C | **-.48** | | .16 | .20 |
| 14 - P | **-.52** | -.21 | .36 | 18 - C | **-.46** | | -.21 | .33 |
| 26 - P | **-.50** | | .24 | 14 - P | **-.43** | -.21 | -.20 | .36 |
| 8 - S (R) | **.47** | | .22 | 4 - S (R) | **.39** | | **.39** | .37 |
| 12 - S (R) | **.45** | .23 | .31 | 28 - S | | **.76** | .18 | .64 |
| 24 - S (R) | **.41** | .19 | .24 | 32 - S | | **.76** | | .58 |
| 31 - C | **-.37** | .10 | .13 | 25 - I | -.11 | **.70** | .19 | .52 |
| 19 - P | -.16 | | .03 | 27 - I | | **.63** | -.14 | .40 |
| 28 - S | | **.78** | .65 | 30 - I | | **.61** | -.30 | .43 |
| 32 - S | | **.77** | .59 | 20 - S | -.19 | **.57** | **.54** | .62 |
| 25 - I | | **.73** | .52 | 23 - I | .26 | **.50** | | .36 |
| 27 - I | | **.60** | .35 | 6 - I | | **.49** | .11 | .27 |
| 20 - S | | **.60** | .40 | 11 - I | | **.42** | -.21 | .21 |
| 30 - I | | **.55** | .29 | 24 - S (R) | | .13 | **.78** | .54 |
| 6 - I | | **.51** | .27 | 8 - S (R) | .24 | | **.47** | .33 |
| 23 - I | .19 | **.48** | .31 | 12 - S (R) | **.31** | .21 | **.31** | .33 |
| 11 - I | | **.39** | .15 | 19 - P | | | -.19 | .05 |

*Note*. *N* = 108 (Polish sample no. 4.) The extraction method was principal axis analysis with direct oblimin rotation. Loadings >|0.30| are in bold, loadings < |0.10| are omitted. Reverse scored items are denoted with an (R). Items No. 1, 5, 9, 15, 16, 17, 21, and 29 were left out due to lack of reliability. Items from Krampen (1991).

**Table A8**

*Results From Explorative Structural Equation Modeling (ESEM) of the I-SEE Questionnaire in the Transcultural Total Sample (N = 1,084)*

| Factor<br>Item | 1 | 2 | <br>Item | 1 | 2 | 3 | 4 |
|---|---|---|---|---|---|---|---|
| 13 - C | **.67** | - | 28 - S | **.71** | - | - | .20 |
| 9 - C | **.60** | .11 | 32 - S | **.67** | - | - | .22 |
| 2 - C | **.58** | .13 | 25 - I | **.57** | - | .22 | - |
| 10 - P | **.58** | - | 20 - S | **.54** | .10 | .18 | - |
| 3 - P | **.57** | - | 30 - I | **.54** | -.29 | **.37** | - |
| 14 - P | **.55** | - | 27 - I | **.53** | -.16 | .19 | - |
| 18 - C | **.53** | - | 6 - I | **.49** | -.12 | - | - |
| 7 - C | **.51** | - | 16 - S | **.47** | - | - | **-.39** |
| 22 - P | **.51** | - | 11 - I | **.44** | - | .12 | **-.40** |
| 15 - C | **.47** | - | 23 - I | **.42** | - | -.15 | -.16 |
| 21 - C | **.46** | - | 1 - I | **.31** | - | - | - |
| 31 - C | **.41** | - | 5 - I | .26 | - | .19 | -.12 |
| 26 - P | **.40** | - | 14 - P | -.11 | **.71** | -.10 | -.15 |
| 29 - P | **.36** | - | 3 - P | -.10 | **.65** | - | - |
| 19 - P | **.32** | .11 | 2 - C | - | **.59** | - | .13 |
| 17 - P | **.31** | -.20 | 13 - C | - | **.59** | .16 | - |
| 28 - S | - | **.77** | 9 - C | -.20 | **.50** | .17 | - |
| 32 - S | - | **.74** | 22 - P | - | **.50** | - | -.11 |
| 25 - I | - | **.63** | 19 - P | .11 | **.41** | - | -.22 |
| 20 - S | - | **.62** | 26 - P | - | **.36** | - | - |
| 6 - I | -.13 | **.49** | 10 - P | - | **.34** | **.34** | - |
| 27 - I | - | **.44** | 15 - C | - | **.32** | .19 | .18 |
| 23 - I | - | **.42** | 29 - P | .10 | .27 | .16 | - |
| 30 - I | - | **.40** | 24 - S (R) | .19 | - | **-.59** | - |
| 16 - S | - | **.37** | 4 - S (R) | .13 | - | **-.52** | - |
| 24 - S (R) | -.29 | **.34** | 21 - C | - | .10 | **.52** | .16 |
| 1 - I | - | .29 | 8 - S (R) | - | - | **-.51** | - |
| 12 - S (R) | **-.50** | .29 | 7 - C | - | .20 | **.46** | .16 |
| 4 - S (R) | **-.36** | .27 | 12 - S (R) | .19 | -.25 | **-.45** | - |
| 5 - I | .14 | .18 | 17 - P | -.10 | - | **.44** | - |
| 8 - S (R) | **-.32** | .17 | 18 - C | - | **.30** | **.35** | .12 |
| 11 - I | - | - | 31 - C | - | .19 | .26 | **.32** |

*Note*. Loadings >|0.30| are in bold, loadings < |0.10| are omitted. Reverse scored items are denoted with an (R). Items from Krampen (1991).

## IMINCE Output: German and Kenyan sample

I M I N C E

Item Measurement Invariance
Based on Exploratory Factor Analysis

Release Version 2.01
August, 2002
University Rovira i Virgili
Tarragona, SPAIN

Programing:
Urbano Lorenzo-Seva

Mathematical Specification:
Urbano Lorenzo-Seva
Pere J. Ferrando

Date: Wednesday, June 25, 2025
Time: 19:34:49

-------------------------------------------------------------------------------

DETAILS OF ANALYSIS

Target sample data file : German sample 2.a
Number of observations in Target sample : 206
Replication sample data file : Kenyan sample
Number of observations in Replication sample : 91

Number of variables : 32
Number of components : 2

Dispersion matrix : Covariances
Method for components extraction : Principal Components Analysis

Rotation to simplicity of Target loading matrix : Normalized Varimax
Rotation to similitude between loading matrices : Orthogonal Procrustes

Bias corrected Percentile intervals obtained by : Bootstrap resampling
Critical values obtained by : Bootstrap resampling
Number of samples in Bootstrap resampling : 5000

Alpha value : 0.05

-------------------------------------------------------------------------------

ITEM DIFFICULTIES

| Item | Target Sample | Replication Sample | Student's t | Effect Size (Cohen's d) |
|---|---|---|---|---|
| 1 | 4.07 | 4.43 | -2.40** | -0.30 |
| 2 | 3.52 | 2.30 | 6.58** | 0.83 |
| 3 | 3.34 | 2.47 | 5.15** | 0.65 |
| 4 | 4.16 | 3.82 | 1.55 | 0.20 |
| 5 | 3.19 | 3.24 | -0.25 | -0.03 |
| 6 | 4.02 | 4.66 | -3.29** | -0.41 |
| 7 | 2.86 | 2.73 | 0.69 | 0.09 |
| 8 | 3.53 | 3.00 | 1.98** | 0.25 |

| | | | | |
|---|---|---|---|---|
| 9 | 3.61 | 1.78 | 11.12** | 1.40 |
| 10 | 2.77 | 2.29 | 2.44** | 0.31 |
| 11 | 4.38 | 4.49 | -0.55 | -0.07 |
| 12 | 3.98 | 4.41 | -1.95 | -0.25 |
| 13 | 2.97 | 1.97 | 5.16** | 0.65 |
| 14 | 3.32 | 1.67 | 8.56** | 1.08 |
| 15 | 2.98 | 2.18 | 3.96** | 0.50 |
| 16 | 4.44 | 4.62 | -0.89 | -0.11 |
| 17 | 1.93 | 2.63 | -1.96** | -0.25 |
| 18 | 2.72 | 2.18 | 2.54** | 0.32 |
| 19 | 4.04 | 2.19 | 8.29** | 1.04 |
| 20 | 3.92 | 3.64 | 1.40 | 0.18 |
| 21 | 2.39 | 2.65 | -1.20 | -0.15 |
| 22 | 3.12 | 2.21 | 4.44** | 0.56 |
| 23 | 4.74 | 3.68 | 5.16** | 0.65 |
| 24 | 3.36 | 3.14 | 0.68 | 0.09 |
| 25 | 4.48 | 4.46 | 0.10 | 0.01 |
| 26 | 3.09 | 2.54 | 2.56** | 0.32 |
| 27 | 4.38 | 4.81 | -1.99** | -0.25 |
| 28 | 4.23 | 4.37 | -0.67 | -0.08 |
| 29 | 3.24 | 2.79 | 2.11** | 0.27 |
| 30 | 3.77 | 4.80 | -4.64** | -0.58 |
| 31 | 3.02 | 2.43 | 2.76** | 0.35 |
| 32 | 4.46 | 4.78 | -1.71 | -0.22 |

**Significant differences

--------------------------------------------------------------------------------

ITEM DISCRIMINATIONS

Item discriminations (Loading Matrix)
Target Sample

| Item | C 1 | C 2 |
|---|---|---|
| 1 | -0.121 | 0.185 |
| 2 | 0.324 | 0.065 |
| 3 | 0.470 | 0.087 |
| 4 | -0.768 | 0.279 |
| 5 | 0.063 | -0.106 |
| 6 | -0.206 | 1.036 |
| 7 | 0.441 | -0.135 |
| 8 | -0.574 | 0.146 |
| 9 | 0.440 | 0.056 |
| 10 | 0.538 | -0.051 |
| 11 | 0.146 | 0.918 |
| 12 | -0.566 | 0.979 |
| 13 | 0.576 | -0.075 |
| 14 | 0.561 | -0.100 |
| 15 | 0.521 | 0.784 |
| 16 | 0.032 | 0.978 |
| 17 | 2.319 | 0.733 |
| 18 | 0.792 | 0.618 |
| 19 | 0.432 | 0.951 |
| 20 | -0.023 | 1.095 |
| 21 | 0.710 | 0.704 |
| 22 | 0.574 | 0.795 |
| 23 | -0.120 | 0.998 |
| 24 | -1.039 | 1.467 |
| 25 | -0.400 | 1.259 |
| 26 | 0.235 | 1.059 |
| 27 | -0.054 | 1.235 |

| | | |
|---|---|---|
| 28 | -0.286 | 1.279 |
| 29 | 0.209 | 0.900 |
| 30 | 0.127 | 1.035 |
| 31 | 0.539 | 0.781 |
| 32 | -0.072 | 0.999 |

Bias-corrected Percentile Intervals of Item discriminations
Target Sample

| Item | C 1 | C 2 |
|---|---|---|
| 1 | ( -0.286; 0.070) | ( 0.088; 0.406) |
| 2 | ( 0.068; 0.606) | ( -0.107; 0.842) |
| 3 | ( 0.249; 0.727) | ( -0.411; 0.269) |
| 4 | ( -1.244; -0.494) | ( 0.067; 0.676) |
| 5 | ( -0.295; 0.350) | ( -0.319; 0.485) |
| 6 | ( -0.550; 0.158) | ( -0.022; 1.876) |
| 7 | ( 0.181; 0.721) | ( -0.526; 0.126) |
| 8 | ( -1.257; -0.172) | ( -0.302; 0.729) |
| 9 | ( 0.170; 0.686) | ( -0.126; 0.653) |
| 10 | ( 0.323; 0.840) | ( -0.287; 0.437) |
| 11 | ( -0.138; 0.521) | ( -0.089; 1.817) |
| 12 | ( -0.882; -0.233) | ( 0.174; 1.883) |
| 13 | ( 0.306; 1.004) | ( -0.482; 0.251) |
| 14 | ( 0.398; 0.762) | ( -0.267; 0.205) |
| 15 | ( 0.167; 0.857) | ( -0.495; 1.559) |
| 16 | ( -0.225; 0.481) | ( -0.129; 1.843) |
| 17 | ( 0.896; 3.348) | ( -2.206; 1.129) |
| 18 | ( 0.399; 1.200) | ( -0.692; 1.407) |
| 19 | ( 0.135; 0.810) | ( -0.171; 1.766) |
| 20 | ( -0.296; 0.395) | ( -0.047; 1.885) |
| 21 | ( 0.409; 1.051) | ( -0.444; 1.438) |
| 22 | ( 0.336; 0.852) | ( -0.177; 1.586) |
| 23 | ( -0.368; 0.298) | ( -0.043; 1.927) |
| 24 | ( -2.399; -0.519) | ( -0.991; 2.100) |
| 25 | ( -0.861; -0.089) | ( 0.058; 2.087) |
| 26 | ( -0.306; 0.583) | ( -0.148; 1.777) |
| 27 | ( -0.575; 0.316) | ( -0.055; 2.041) |
| 28 | ( -0.754; 0.098) | ( -0.120; 2.058) |
| 29 | ( -0.145; 0.535) | ( -0.039; 1.701) |
| 30 | ( -0.194; 0.600) | ( -0.170; 1.821) |
| 31 | ( 0.210; 0.874) | ( -0.406; 1.567) |
| 32 | ( -0.329; 0.376) | ( -0.072; 1.878) |

Item discriminations (Loading Matrix)
Replication Sample

| Item | C 1 | C 2 |
|---|---|---|
| 1 | -0.275 | -0.082 |
| 2 | -0.101 | 0.084 |
| 3 | 0.015 | 0.251 |
| 4 | -0.395 | -0.114 |
| 5 | 0.350 | -0.020 |
| 6 | -0.139 | 0.002 |
| 7 | 1.395 | 0.341 |
| 8 | 1.508 | -0.161 |
| 9 | 1.159 | 0.247 |
| 10 | 1.290 | 0.445 |
| 11 | 1.395 | 0.141 |
| 12 | 1.176 | 0.061 |
| 13 | 1.316 | 0.390 |

| | | |
|---|---|---|
| 14 | 1.261 | 0.293 |
| 15 | 1.294 | 0.452 |
| 16 | 1.427 | 0.271 |
| 17 | 1.324 | 0.355 |
| 18 | 1.337 | 0.413 |
| 19 | 1.127 | 1.368 |
| 20 | -0.068 | -0.013 |
| 21 | -0.251 | 1.356 |
| 22 | 0.006 | 1.339 |
| 23 | -0.193 | -0.002 |
| 24 | -0.276 | 1.179 |
| 25 | -0.215 | -0.026 |
| 26 | 0.045 | 1.396 |
| 27 | -0.309 | 1.551 |
| 28 | -0.438 | 1.523 |
| 29 | 0.166 | 1.492 |
| 30 | -0.326 | 1.494 |
| 31 | 0.051 | 1.394 |
| 32 | -0.348 | 1.484 |

Bias-corrected Percentile Intervals of Item discriminations
Replication Sample

| Item | C 1 | C 2 |
|---|---|---|
| 1 | ( -0.601; 0.152) | ( -0.343; 0.734) |
| 2 | ( -0.585; 0.918) | ( -0.836; 0.801) |
| 3 | ( -0.387; 0.955) | ( -0.892; 0.799) |
| 4 | ( -1.244; 0.238) | ( -0.929; 1.030) |
| 5 | ( -0.476; 0.596) | ( -0.336; 0.921) |
| 6 | ( -0.480; 0.580) | ( -0.496; 0.643) |
| 7 | ( 0.350; 2.447) | ( -0.126; 1.108) |
| 8 | ( -1.200; 2.814) | ( -1.184; 2.697) |
| 9 | ( 0.086; 2.229) | ( 0.004; 0.828) |
| 10 | ( 0.336; 2.307) | ( 0.056; 1.061) |
| 11 | ( -0.776; 2.603) | ( -0.456; 1.589) |
| 12 | ( -0.964; 2.546) | ( -0.586; 1.590) |
| 13 | ( 0.393; 2.294) | ( -0.103; 1.133) |
| 14 | ( 0.042; 2.244) | ( -0.472; 1.304) |
| 15 | ( 0.094; 2.327) | ( 0.031; 0.908) |
| 16 | ( -0.683; 2.615) | ( -0.278; 1.590) |
| 17 | ( 0.058; 2.390) | ( 0.048; 0.796) |
| 18 | ( 0.349; 2.354) | ( 0.004; 1.003) |
| 19 | ( -0.394; 2.121) | ( -0.208; 2.467) |
| 20 | ( -0.936; 0.410) | ( -0.596; 0.986) |
| 21 | ( -0.572; 0.657) | ( -0.466; 2.463) |
| 22 | ( -0.425; 0.464) | ( -0.243; 2.410) |
| 23 | ( -0.796; 0.857) | ( -0.615; 1.001) |
| 24 | ( -1.088; 0.228) | ( -0.732; 2.465) |
| 25 | ( -0.567; 0.607) | ( -0.483; 0.728) |
| 26 | ( -0.384; 0.352) | ( -0.069; 2.482) |
| 27 | ( -0.671; 0.834) | ( -0.561; 2.897) |
| 28 | ( -0.770; 0.714) | ( -0.597; 2.802) |
| 29 | ( -0.314; 0.498) | ( -0.039; 2.570) |
| 30 | ( -0.607; 0.700) | ( -0.445; 2.879) |
| 31 | ( -0.442; 0.400) | ( -0.116; 2.454) |
| 32 | ( -0.656; 0.735) | ( -0.495; 2.853) |

Difference Values of Item discriminations

| Item | C 1 | C 2 |
|---|---|---|

| | | |
|---|---|---|
| 1 | 0.154 | 0.268 |
| 2 | 0.426 | -0.019 |
| 3 | 0.456 | -0.165 |
| 4 | -0.373 | 0.394 |
| 5 | -0.287 | -0.086 |
| 6 | -0.067 | 1.034 |
| 7 | -0.954 | -0.475 |
| 8 | -2.082 | 0.307 |
| 9 | -0.719 | -0.192 |
| 10 | -0.752 | -0.496 |
| 11 | -1.248 | 0.777 |
| 12 | -1.742 | 0.917 |
| 13 | -0.741 | -0.465 |
| 14 | -0.700 | -0.394 |
| 15 | -0.773 | 0.332 |
| 16 | -1.395 | 0.707 |
| 17 | 0.996 | 0.379 |
| 18 | -0.545 | 0.205 |
| 19 | -0.696 | -0.417 |
| 20 | 0.044 | 1.107 |
| 21 | 0.961 | -0.652 |
| 22 | 0.568 | -0.544 |
| 23 | 0.074 | 1.000 |
| 24 | -0.763 | 0.289 |
| 25 | -0.185 | 1.285 |
| 26 | 0.190 | -0.337 |
| 27 | 0.255 | -0.316 |
| 28 | 0.152 | -0.245 |
| 29 | 0.044 | -0.592 |
| 30 | 0.453 | -0.459 |
| 31 | 0.487 | -0.612 |
| 32 | 0.276 | -0.485 |

** Significant differences

--------------------------------------------------------------------------------

ITEM RESIDUAL VARIANCES

Residual Variances
Target Sample

| Item | Residual Variance |
|---|---|
| 1 | 0.956 |
| 2 | 2.072 |
| 3 | 1.355 |
| 4 | 2.105 |
| 5 | 2.711 |
| 6 | 1.583 |
| 7 | 1.423 |
| 8 | 2.985 |
| 9 | 1.167 |
| 10 | 1.787 |
| 11 | 1.024 |
| 12 | 1.149 |
| 13 | 1.779 |
| 14 | 1.309 |
| 15 | 1.294 |
| 16 | 1.026 |
| 17 | 3.976 |
| 18 | 1.646 |

| | |
|---|---|
| 19 | 1.287 |
| 20 | 1.557 |
| 21 | 1.530 |
| 22 | 1.307 |
| 23 | 1.475 |
| 24 | 4.504 |
| 25 | 0.776 |
| 26 | 1.476 |
| 27 | 1.106 |
| 28 | 0.905 |
| 29 | 1.670 |
| 30 | 1.946 |
| 31 | 1.555 |
| 32 | 0.740 |

Bias-corrected Percentile Intervals of Residual Variances
Target Sample

| Item | Residual Variance Interval |
|---|---|
| 1 | ( 0.717; 1.186) |
| 2 | ( 1.116; 3.641) |
| 3 | ( 1.065; 1.597) |
| 4 | ( 1.217; 3.519) |
| 5 | ( 1.698; 4.208) |
| 6 | ( 0.671; 3.331) |
| 7 | ( 1.083; 1.675) |
| 8 | ( 1.404; 4.740) |
| 9 | ( 0.892; 1.395) |
| 10 | ( 0.978; 3.113) |
| 11 | ( 0.766; 1.236) |
| 12 | ( 0.709; 1.552) |
| 13 | ( 0.969; 3.055) |
| 14 | ( 1.085; 1.549) |
| 15 | ( 0.944; 1.543) |
| 16 | ( 0.748; 1.322) |
| 17 | ( 0.112; 6.206) |
| 18 | ( 1.055; 2.058) |
| 19 | ( 1.043; 1.659) |
| 20 | ( 0.725; 3.406) |
| 21 | ( 0.743; 2.833) |
| 22 | ( 1.044; 1.584) |
| 23 | ( 0.432; 3.355) |
| 24 | ( 0.654; 6.194) |
| 25 | ( 0.434; 1.233) |
| 26 | ( 0.919; 2.008) |
| 27 | ( 0.608; 1.712) |
| 28 | ( 0.503; 1.580) |
| 29 | ( 0.869; 2.855) |
| 30 | ( 1.079; 3.663) |
| 31 | ( 1.193; 1.854) |
| 32 | ( 0.488; 1.009) |

Residual Variances
Replication Sample

| Item | Residual Variance |
|---|---|
| 1 | 2.294 |
| 2 | 2.147 |
| 3 | 2.208 |

| | |
|---|---|
| 4 | 2.943 |
| 5 | 2.939 |
| 6 | 1.678 |
| 7 | 1.939 |
| 8 | 5.173 |
| 9 | 1.097 |
| 10 | 1.617 |
| 11 | 2.021 |
| 12 | 2.941 |
| 13 | 1.136 |
| 14 | 2.238 |
| 15 | 1.608 |
| 16 | 1.621 |
| 17 | 1.785 |
| 18 | 1.615 |
| 19 | 1.778 |
| 20 | 1.985 |
| 21 | 1.622 |
| 22 | 1.670 |
| 23 | 2.993 |
| 24 | 2.767 |
| 25 | 1.345 |
| 26 | 1.529 |
| 27 | 1.145 |
| 28 | 1.150 |
| 29 | 1.493 |
| 30 | 1.050 |
| 31 | 1.970 |
| 32 | 1.123 |

Bias-corrected Percentile Intervals of Residual Variances
Replication Sample

| Item | Residual Variance Interval |
|---|---|
| 1 | ( 1.726; 2.993) |
| 2 | ( 1.676; 3.036) |
| 3 | ( 1.739; 2.927) |
| 4 | ( 2.350; 3.625) |
| 5 | ( 2.455; 3.582) |
| 6 | ( 1.203; 2.436) |
| 7 | ( 1.508; 2.669) |
| 8 | ( 3.247; 9.378) |
| 9 | ( 0.758; 1.656) |
| 10 | ( 1.164; 2.411) |
| 11 | ( 1.587; 2.983) |
| 12 | ( 2.110; 4.088) |
| 13 | ( 0.824; 1.856) |
| 14 | ( 0.845; 5.603) |
| 15 | ( 1.099; 2.221) |
| 16 | ( 1.194; 2.765) |
| 17 | ( 1.282; 2.315) |
| 18 | ( 1.187; 2.214) |
| 19 | ( 1.381; 2.922) |
| 20 | ( 1.575; 2.699) |
| 21 | ( 1.278; 2.257) |
| 22 | ( 1.292; 2.261) |
| 23 | ( 2.556; 3.788) |
| 24 | ( 2.242; 3.821) |
| 25 | ( 1.057; 2.045) |
| 26 | ( 1.119; 2.077) |
| 27 | ( 0.733; 1.975) |

| | | |
|---|---|---|
| 28 | ( 0.705; | 1.939) |
| 29 | ( 1.049; | 2.080) |
| 30 | ( 0.745; | 1.688) |
| 31 | ( 1.482; | 2.612) |
| 32 | ( 0.752; | 1.923) |

Difference Values in Residual Variances

| Item | Residual Variance |
|---|---|
| 1 | -1.338** |
| 2 | -0.075 |
| 3 | -0.853** |
| 4 | -0.838 |
| 5 | -0.228 |
| 6 | -0.095 |
| 7 | -0.517 |
| 8 | -2.188 |
| 9 | 0.070 |
| 10 | 0.170 |
| 11 | -0.998** |
| 12 | -1.793** |
| 13 | 0.642 |
| 14 | -0.929 |
| 15 | -0.314 |
| 16 | -0.594 |
| 17 | 2.191 |
| 18 | 0.032 |
| 19 | -0.492 |
| 20 | -0.428 |
| 21 | -0.092 |
| 22 | -0.363 |
| 23 | -1.518 |
| 24 | 1.736 |
| 25 | -0.569 |
| 26 | -0.054 |
| 27 | -0.038 |
| 28 | -0.245 |
| 29 | 0.177 |
| 30 | 0.896 |
| 31 | -0.414 |
| 32 | -0.384 |

** Significant differences

-------------------------------------------------------------------------------

OVERALL INDICES OF ITEM MEASUREMENT INVARIANCE

ITEM DIFFICULTIES

Hotteling's T Square

TSQ = 346.838053; Freedom degrees = 32 and 264; F = 9.699708 (P = 0.00000)

-------------------------------------------------------------------------------

OVERALL INDICES OF ITEM MEASUREMENT INVARIANCE

CONGRUENCE INDICES

Overall Fit Indices Per Item

| Item | Observed Congruence | Critical value at alpha = 0.05 |
|---|---|---|
| 1 | 0.282 | -0.960 |
| 2 | -0.630** | -0.092 |
| 3 | 0.238 | -0.008 |
| 4 | 0.807 | 0.551 |
| 5 | 0.560 | -0.985 |
| 6 | 0.209** | 0.771 |
| 7 | 0.860 | -0.178 |
| 8 | -0.990** | 0.456 |
| 9 | 0.996 | -0.011 |
| 10 | 0.910 | 0.352 |
| 11 | 0.256** | 0.561 |
| 12 | -0.455** | -0.035 |
| 13 | 0.914 | -0.071 |
| 14 | 0.919 | 0.718 |
| 15 | 0.797 | 0.117 |
| 16 | 0.219** | 0.449 |
| 17 | 0.999 | 0.961 |
| 18 | 0.935 | 0.223 |
| 19 | 0.966 | 0.604 |
| 20 | -0.164** | 0.486 |
| 21 | 0.563 | 0.538 |
| 22 | 0.813 | 0.466 |
| 23 | 0.109** | 0.236 |
| 24 | 0.926 | 0.848 |
| 25 | 0.186** | 0.548 |
| 26 | 0.983 | -0.016 |
| 27 | 0.988 | -0.760 |
| 28 | 0.998 | 0.731 |
| 29 | 0.993 | 0.597 |
| 30 | 0.944 | 0.566 |
| 31 | 0.844 | 0.545 |
| 32 | 0.987 | -0.729 |

** Significant differences

Overall Fit Indices Per Component

| Component | Observed Congruence | Critical value at alpha = 0.05 |
|---|---|---|
| 1 | 0.759 | 0.660 |
| 2 | 0.480** | 0.609 |

** Significant differences

Overall Fit Index

| Observed Congruence | Critical value at alpha = 0.05 |
|---|---|
| 0.633** | 0.663 |

** Significant differences

DISCREPANCY INDICES

Overall Fit Indices Per Item

| Item | Observed Similitude | Critical value at alpha = 0.05 |
|---|---|---|
| 1 | 0.095 | 0.454 |
| 2 | 0.182 | 0.815 |
| 3 | 0.235 | 0.843 |
| 4 | 0.294 | 0.950 |
| 5 | 0.090 | 0.821 |
| 6 | 1.074 | 1.103 |
| 7 | 1.135** | 0.418 |
| 8 | 4.429** | 1.875 |
| 9 | 0.554 | 0.646 |
| 10 | 0.812** | 0.489 |
| 11 | 2.162** | 1.110 |
| 12 | 3.877** | 2.925 |
| 13 | 0.765 | 1.053 |
| 14 | 0.645** | 0.556 |
| 15 | 0.708 | 1.259 |
| 16 | 2.445** | 1.219 |
| 17 | 1.135 | 1.190 |
| 18 | 0.339 | 1.667 |
| 19 | 0.658 | 1.776 |
| 20 | 1.228 | 1.324 |
| 21 | 1.348 | 1.480 |
| 22 | 0.619 | 1.136 |
| 23 | 1.004 | 1.559 |
| 24 | 0.666 | 2.036 |
| 25 | 1.686 | 1.797 |
| 26 | 0.149 | 1.793 |
| 27 | 0.165 | 2.512 |
| 28 | 0.083 | 1.642 |
| 29 | 0.353 | 1.050 |
| 30 | 0.417 | 1.368 |
| 31 | 0.612 | 1.113 |
| 32 | 0.311 | 1.999 |

** Significant differences

Overall Fit Indices Per Component

| Component | Observed Discrepancy | Critical value at alpha = 0.05 |
|---|---|---|
| 1 | 10.846 | 16.371 |
| 2 | 19.430** | 17.166 |

** Significant differences

Overall Fit Index

| Observed Discrepancy | Critical value at alpha = 0.05 |
|---|---|
| 30.276** | 27.922 |

** Significant differences

--------------------------------------------------------------------------------

IMINCE completed

Computing time: 1.03333333 minutes.

## IMINCE Output: German and Polish sample

I M I N C E

Item Measurement Invariance
Based on Exploratory Factor Analysis

Release Version 2.01
August, 2002
University Rovira i Virgili
Tarragona, SPAIN

Programing:
Urbano Lorenzo-Seva

Mathematical Specification:
Urbano Lorenzo-Seva
Pere J. Ferrando

Date: Wednesday, June 25, 2025
Time: 20:3:32

------------------------------------------------------------------------------

DETAILS OF ANALYSIS

Target sample data file : German sample 2.a
Number of observations in Target sample : 206
Replication sample data file : Polish sample
Number of observations in Replication sample : 108

Number of variables : 32
Number of components : 2

Dispersion matrix : Covariances
Method for components extraction : Principal Components Analysis

Rotation to simplicity of Target loading matrix : Normalized Varimax
Rotation to similitude between loading matrices : Orthogonal Procrustes

Bias corrected Percentile intervals obtained by : Bootstrap resampling
Critical values obtained by : Bootstrap resampling
Number of samples in Bootstrap resampling : 5000

Alpha value : 0.05

------------------------------------------------------------------------------

ITEM DIFFICULTIES

| Item | Target Sample | Replication Sample | Student's t | Effect Size (Cohen's d) |
|---|---|---|---|---|
| 1 | 4.07 | 4.08 | -0.10 | -0.01 |
| 2 | 3.52 | 3.24 | 1.66 | 0.20 |
| 3 | 3.34 | 3.21 | 0.83 | 0.10 |
| 4 | 4.16 | 3.01 | 6.05** | 0.72 |
| 5 | 3.19 | 2.80 | 1.79 | 0.21 |
| 6 | 4.02 | 4.08 | -0.36 | -0.04 |
| 7 | 2.86 | 3.85 | -6.33** | -0.75 |

| | | | | |
|---|---|---|---|---|
| 8 | 3.53 | 2.91 | 3.09** | 0.37 |
| 9 | 3.61 | 3.90 | -1.67 | -0.20 |
| 10 | 2.77 | 3.10 | -1.93 | -0.23 |
| 11 | 4.38 | 4.26 | 0.76 | 0.09 |
| 12 | 3.98 | 3.89 | 0.51 | 0.06 |
| 13 | 2.97 | 2.94 | 0.21 | 0.02 |
| 14 | 3.32 | 2.86 | 3.03** | 0.36 |
| 15 | 2.98 | 3.06 | -0.47 | -0.06 |
| 16 | 4.44 | 4.28 | 1.00 | 0.12 |
| 17 | 1.93 | 3.13 | -3.82** | -0.45 |
| 18 | 2.72 | 3.36 | -3.40** | -0.40 |
| 19 | 4.04 | 4.12 | -0.47 | -0.06 |
| 20 | 3.92 | 3.76 | 0.87 | 0.10 |
| 21 | 2.39 | 3.44 | -6.07** | -0.72 |
| 22 | 3.12 | 3.23 | -0.66 | -0.08 |
| 23 | 4.74 | 4.95 | -1.15 | -0.14 |
| 24 | 3.36 | 2.95 | 1.43 | 0.17 |
| 25 | 4.48 | 4.71 | -1.39 | -0.17 |
| 26 | 3.09 | 3.16 | -0.35 | -0.04 |
| 27 | 4.38 | 4.72 | -2.00** | -0.24 |
| 28 | 4.23 | 4.41 | -0.93 | -0.11 |
| 29 | 3.24 | 4.43 | -6.90** | -0.82 |
| 30 | 3.77 | 4.75 | -4.89** | -0.58 |
| 31 | 3.02 | 3.64 | -3.09** | -0.37 |
| 32 | 4.46 | 4.44 | 0.08 | 0.01 |

**Significant differences

-------------------------------------------------------------------------------

ITEM DISCRIMINATIONS

Item discriminations (Loading Matrix)
Target Sample

| Item | C 1 | C 2 |
|---|---|---|
| 1 | -0.121 | 0.185 |
| 2 | 0.324 | 0.065 |
| 3 | 0.470 | 0.087 |
| 4 | -0.768 | 0.279 |
| 5 | 0.063 | -0.106 |
| 6 | -0.206 | 1.036 |
| 7 | 0.441 | -0.135 |
| 8 | -0.574 | 0.146 |
| 9 | 0.440 | 0.056 |
| 10 | 0.538 | -0.051 |
| 11 | 0.146 | 0.918 |
| 12 | -0.566 | 0.979 |
| 13 | 0.576 | -0.075 |
| 14 | 0.561 | -0.100 |
| 15 | 0.521 | 0.784 |
| 16 | 0.032 | 0.978 |
| 17 | 2.319 | 0.733 |
| 18 | 0.792 | 0.618 |
| 19 | 0.432 | 0.951 |
| 20 | -0.023 | 1.095 |
| 21 | 0.710 | 0.704 |
| 22 | 0.574 | 0.795 |
| 23 | -0.120 | 0.998 |
| 24 | -1.039 | 1.467 |
| 25 | -0.400 | 1.259 |
| 26 | 0.235 | 1.059 |

| | | |
|---|---|---|
| 27 | -0.054 | 1.235 |
| 28 | -0.286 | 1.279 |
| 29 | 0.209 | 0.900 |
| 30 | 0.127 | 1.035 |
| 31 | 0.539 | 0.781 |
| 32 | -0.072 | 0.999 |

Bias-corrected Percentile Intervals of Item discriminations
Target Sample

| Item | C 1 | C 2 |
|---|---|---|
| 1 | ( -0.288; 0.071) | ( 0.095; 0.426) |
| 2 | ( 0.068; 0.611) | ( -0.109; 0.842) |
| 3 | ( 0.261; 0.739) | ( -0.430; 0.263) |
| 4 | ( -1.281; -0.500) | ( 0.084; 0.700) |
| 5 | ( -0.297; 0.355) | ( -0.318; 0.478) |
| 6 | ( -0.558; 0.150) | ( -0.019; 1.882) |
| 7 | ( 0.184; 0.708) | ( -0.530; 0.122) |
| 8 | ( -1.200; -0.169) | ( -0.317; 0.708) |
| 9 | ( 0.175; 0.700) | ( -0.133; 0.644) |
| 10 | ( 0.321; 0.864) | ( -0.294; 0.454) |
| 11 | ( -0.135; 0.518) | ( -0.092; 1.808) |
| 12 | ( -0.875; -0.223) | ( 0.177; 1.885) |
| 13 | ( 0.319; 1.004) | ( -0.489; 0.227) |
| 14 | ( 0.392; 0.767) | ( -0.274; 0.192) |
| 15 | ( 0.167; 0.871) | ( -0.493; 1.575) |
| 16 | ( -0.225; 0.460) | ( -0.130; 1.852) |
| 17 | ( 0.887; 3.351) | ( -2.154; 1.143) |
| 18 | ( 0.401; 1.195) | ( -0.700; 1.397) |
| 19 | ( 0.129; 0.794) | ( -0.180; 1.758) |
| 20 | ( -0.305; 0.380) | ( -0.049; 1.897) |
| 21 | ( 0.407; 1.060) | ( -0.440; 1.426) |
| 22 | ( 0.337; 0.863) | ( -0.166; 1.594) |
| 23 | ( -0.363; 0.295) | ( -0.047; 1.924) |
| 24 | ( -2.394; -0.537) | ( -0.827; 2.139) |
| 25 | ( -0.863; -0.096) | ( 0.069; 2.114) |
| 26 | ( -0.310; 0.568) | ( -0.134; 1.809) |
| 27 | ( -0.548; 0.321) | ( -0.057; 2.068) |
| 28 | ( -0.742; 0.091) | ( -0.114; 2.091) |
| 29 | ( -0.141; 0.551) | ( -0.040; 1.694) |
| 30 | ( -0.191; 0.578) | ( -0.168; 1.835) |
| 31 | ( 0.206; 0.877) | ( -0.408; 1.578) |
| 32 | ( -0.326; 0.368) | ( -0.070; 1.868) |

Item discriminations (Loading Matrix)
Replication Sample

| Item | C 1 | C 2 |
|---|---|---|
| 1 | 0.058 | -0.212 |
| 2 | 0.693 | -0.216 |
| 3 | 0.922 | -0.233 |
| 4 | -0.792 | 0.228 |
| 5 | -0.939 | -1.757 |
| 6 | -0.327 | 0.171 |
| 7 | 0.874 | -0.133 |
| 8 | -0.683 | 0.252 |
| 9 | 0.478 | -0.584 |
| 10 | 0.900 | -0.269 |
| 11 | -0.134 | 0.199 |
| 12 | -0.753 | 0.603 |

| | | |
|---|---|---|
| 13 | 1.037 | -0.407 |
| 14 | 0.719 | -0.270 |
| 15 | 0.099 | -0.192 |
| 16 | -0.224 | 0.128 |
| 17 | 0.638 | -0.091 |
| 18 | 0.782 | -0.340 |
| 19 | 0.115 | -0.388 |
| 20 | -0.452 | 0.223 |
| 21 | 0.399 | -0.067 |
| 22 | 0.954 | -0.029 |
| 23 | -0.742 | -0.575 |
| 24 | -0.794 | 0.158 |
| 25 | -0.267 | 0.202 |
| 26 | 0.610 | 0.041 |
| 27 | -0.189 | 0.117 |
| 28 | -0.386 | 0.444 |
| 29 | -0.120 | 0.068 |
| 30 | -0.027 | -0.253 |
| 31 | 0.388 | 0.688 |
| 32 | -0.298 | 0.314 |

Bias-corrected Percentile Intervals of Item discriminations
Replication Sample

| Item | C 1 | C 2 |
|---|---|---|
| 1 | ( -0.410; 1.367) | ( -2.675; 0.300) |
| 2 | ( 0.408; 0.912) | ( -0.652; 0.124) |
| 3 | ( 0.707; 1.103) | ( -0.562; -0.041) |
| 4 | ( -1.093; -0.463) | ( -0.396; 0.734) |
| 5 | ( -1.744; -0.026) | ( -2.728; -0.243) |
| 6 | ( -0.605; -0.008) | ( -0.446; 0.673) |
| 7 | ( 0.564; 1.118) | ( -0.667; 0.323) |
| 8 | ( -1.002; -0.252) | ( -0.325; 0.816) |
| 9 | ( -0.003; 1.059) | ( -3.074; -0.212) |
| 10 | ( 0.624; 1.149) | ( -0.750; 0.243) |
| 11 | ( -0.573; 0.334) | ( -0.901; 0.905) |
| 12 | ( -1.010; -0.405) | ( 0.374; 0.990) |
| 13 | ( 0.834; 1.231) | ( -0.722; -0.107) |
| 14 | ( 0.488; 0.897) | ( -0.629; 0.028) |
| 15 | ( -0.693; 0.559) | ( -1.998; 0.686) |
| 16 | ( -0.498; 0.093) | ( -0.261; 0.576) |
| 17 | ( 0.420; 0.847) | ( -0.354; 0.195) |
| 18 | ( 0.372; 1.077) | ( -0.891; 0.154) |
| 19 | ( -0.256; 0.422) | ( -0.906; -0.074) |
| 20 | ( -0.747; -0.047) | ( -0.486; 0.799) |
| 21 | ( 0.062; 0.708) | ( -0.382; 0.299) |
| 22 | ( 0.744; 1.182) | ( -0.307; 0.324) |
| 23 | ( -1.908; -0.274) | ( -2.309; 0.132) |
| 24 | ( -1.096; -0.435) | ( -0.345; 0.712) |
| 25 | ( -0.550; 0.017) | ( -0.491; 0.661) |
| 26 | ( 0.187; 1.034) | ( -0.406; 2.374) |
| 27 | ( -0.439; 0.110) | ( -0.484; 0.534) |
| 28 | ( -0.866; 0.069) | ( -0.413; 2.604) |
| 29 | ( -0.374; 0.193) | ( -0.504; 0.613) |
| 30 | ( -0.347; 0.845) | ( -2.641; 0.398) |
| 31 | ( -0.465; 0.814) | ( 0.064; 2.932) |
| 32 | ( -0.630; 0.088) | ( -0.595; 1.013) |

Difference Values of Item discriminations

| Item | C 1 | C 2 |
|---|---|---|
| 1 | -0.179 | 0.398 |
| 2 | -0.369 | 0.281 |
| 3 | -0.452 | 0.319 |
| 4 | 0.025 | 0.051 |
| 5 | 1.002 | 1.651 |
| 6 | 0.121 | 0.865 |
| 7 | -0.433 | -0.001 |
| 8 | 0.108 | -0.106 |
| 9 | -0.038 | 0.640** |
| 10 | -0.362 | 0.218 |
| 11 | 0.280 | 0.719 |
| 12 | 0.187 | 0.375 |
| 13 | -0.462 | 0.332 |
| 14 | -0.158 | 0.169 |
| 15 | 0.421 | 0.976 |
| 16 | 0.256 | 0.850 |
| 17 | 1.681** | 0.824 |
| 18 | 0.011 | 0.958 |
| 19 | 0.317 | 1.339 |
| 20 | 0.429 | 0.872 |
| 21 | 0.311 | 0.771 |
| 22 | -0.380 | 0.824 |
| 23 | 0.622 | 1.572 |
| 24 | -0.245 | 1.309 |
| 25 | -0.133 | 1.057 |
| 26 | -0.375 | 1.018 |
| 27 | 0.135 | 1.118 |
| 28 | 0.100 | 0.835 |
| 29 | 0.329 | 0.832 |
| 30 | 0.155 | 1.288 |
| 31 | 0.151 | 0.093 |
| 32 | 0.226 | 0.685 |

** Significant differences

--------------------------------------------------------------------------------

ITEM RESIDUAL VARIANCES

Residual Variances
Target Sample

| Item | Residual Variance |
|---|---|
| 1 | 0.956 |
| 2 | 2.072 |
| 3 | 1.355 |
| 4 | 2.105 |
| 5 | 2.711 |
| 6 | 1.583 |
| 7 | 1.423 |
| 8 | 2.985 |
| 9 | 1.167 |
| 10 | 1.787 |
| 11 | 1.024 |
| 12 | 1.149 |
| 13 | 1.779 |
| 14 | 1.309 |
| 15 | 1.294 |
| 16 | 1.026 |
| 17 | 3.976 |

| | |
|---|---|
| 18 | 1.646 |
| 19 | 1.287 |
| 20 | 1.557 |
| 21 | 1.530 |
| 22 | 1.307 |
| 23 | 1.475 |
| 24 | 4.504 |
| 25 | 0.776 |
| 26 | 1.476 |
| 27 | 1.106 |
| 28 | 0.905 |
| 29 | 1.670 |
| 30 | 1.946 |
| 31 | 1.555 |
| 32 | 0.740 |

Bias-corrected Percentile Intervals of Residual Variances
Target Sample

| Item | Residual Variance Interval |
|---|---|
| 1 | ( 0.727; 1.196) |
| 2 | ( 1.116; 3.661) |
| 3 | ( 1.064; 1.601) |
| 4 | ( 1.214; 3.457) |
| 5 | ( 1.701; 4.274) |
| 6 | ( 0.669; 3.277) |
| 7 | ( 1.076; 1.669) |
| 8 | ( 1.417; 4.773) |
| 9 | ( 0.883; 1.387) |
| 10 | ( 0.973; 3.146) |
| 11 | ( 0.769; 1.233) |
| 12 | ( 0.706; 1.553) |
| 13 | ( 0.979; 3.017) |
| 14 | ( 1.091; 1.556) |
| 15 | ( 0.952; 1.536) |
| 16 | ( 0.747; 1.317) |
| 17 | ( 0.116; 6.178) |
| 18 | ( 1.059; 2.057) |
| 19 | ( 1.039; 1.653) |
| 20 | ( 0.718; 3.419) |
| 21 | ( 0.747; 2.792) |
| 22 | ( 1.052; 1.578) |
| 23 | ( 0.435; 3.407) |
| 24 | ( 0.773; 6.223) |
| 25 | ( 0.421; 1.233) |
| 26 | ( 0.918; 2.004) |
| 27 | ( 0.610; 1.704) |
| 28 | ( 0.499; 1.575) |
| 29 | ( 0.866; 2.804) |
| 30 | ( 1.075; 3.634) |
| 31 | ( 1.194; 1.850) |
| 32 | ( 0.494; 1.006) |

Residual Variances
Replication Sample

| Item | Residual Variance |
|---|---|
| 1 | 2.843 |
| 2 | 1.118 |

| | |
|---|---|
| 3 | 0.911 |
| 4 | 1.422 |
| 5 | 0.768 |
| 6 | 1.107 |
| 7 | 1.159 |
| 8 | 1.592 |
| 9 | 2.873 |
| 10 | 1.173 |
| 11 | 1.912 |
| 12 | 1.150 |
| 13 | 0.763 |
| 14 | 0.938 |
| 15 | 3.162 |
| 16 | 1.338 |
| 17 | 1.086 |
| 18 | 1.449 |
| 19 | 1.424 |
| 20 | 1.262 |
| 21 | 1.194 |
| 22 | 0.970 |
| 23 | 1.644 |
| 24 | 1.482 |
| 25 | 0.796 |
| 26 | 2.721 |
| 27 | 0.818 |
| 28 | 2.285 |
| 29 | 1.226 |
| 30 | 2.474 |
| 31 | 2.959 |
| 32 | 1.078 |

Bias-corrected Percentile Intervals of Residual Variances
Replication Sample

| Item | Residual Variance Interval |
|---|---|
| 1 | ( 1.158; 5.218) |
| 2 | ( 0.869; 1.506) |
| 3 | ( 0.687; 1.198) |
| 4 | ( 1.164; 2.030) |
| 5 | ( 0.306; 1.128) |
| 6 | ( 0.861; 1.692) |
| 7 | ( 0.857; 1.721) |
| 8 | ( 1.251; 2.258) |
| 9 | ( 1.311; 5.504) |
| 10 | ( 0.914; 1.655) |
| 11 | ( 1.668; 2.476) |
| 12 | ( 0.753; 1.518) |
| 13 | ( 0.568; 1.020) |
| 14 | ( 0.702; 1.248) |
| 15 | ( 1.628; 6.197) |
| 16 | ( 1.049; 1.868) |
| 17 | ( 0.847; 1.402) |
| 18 | ( 1.126; 2.092) |
| 19 | ( 1.033; 1.860) |
| 20 | ( 0.948; 1.797) |
| 21 | ( 0.941; 1.639) |
| 22 | ( 0.733; 1.281) |
| 23 | ( 0.368; 2.905) |
| 24 | ( 1.168; 2.077) |
| 25 | ( 0.610; 1.300) |
| 26 | ( 1.163; 6.231) |

| | | | |
|---|---|---|---|
| 27 | ( | 0.655; | 1.222) |
| 28 | ( | 0.706; | 4.996) |
| 29 | ( | 0.966; | 1.789) |
| 30 | ( | 0.733; | 5.808) |
| 31 | ( | 1.241; | 5.095) |
| 32 | ( | 0.824; | 1.607) |

Difference Values in Residual Variances

| Item | Residual Variance |
|---|---|
| 1 | -1.887 |
| 2 | 0.954 |
| 3 | 0.444 |
| 4 | 0.683 |
| 5 | 1.943** |
| 6 | 0.476 |
| 7 | 0.264 |
| 8 | 1.393 |
| 9 | -1.706 |
| 10 | 0.614 |
| 11 | -0.889** |
| 12 | -0.001 |
| 13 | 1.015 |
| 14 | 0.371 |
| 15 | -1.868** |
| 16 | -0.311 |
| 17 | 2.890 |
| 18 | 0.198 |
| 19 | -0.137 |
| 20 | 0.295 |
| 21 | 0.336 |
| 22 | 0.337 |
| 23 | -0.169 |
| 24 | 3.022 |
| 25 | -0.021 |
| 26 | -1.246 |
| 27 | 0.288 |
| 28 | -1.379 |
| 29 | 0.444 |
| 30 | -0.529 |
| 31 | -1.403 |
| 32 | -0.339 |

** Significant differences

-------------------------------------------------------------------------------

OVERALL INDICES OF ITEM MEASUREMENT INVARIANCE

ITEM DIFFICULTIES

Hotteling's T Square

TSQ = 196.314082; Freedom degrees = 32 and 281; F = 5.525266 (P = 0.00000)

-------------------------------------------------------------------------------

OVERALL INDICES OF ITEM MEASUREMENT INVARIANCE

CONGRUENCE INDICES

Overall Fit Indices Per Item

| Item | Observed Congruence | Critical value at alpha = 0.05 |
|---|---|---|
| 1 | -0.952** | -0.855 |
| 2 | 0.878 | -0.244 |
| 3 | 0.909 | 0.362 |
| 4 | 0.998 | 0.740 |
| 5 | 0.516 | -0.968 |
| 6 | 0.628** | 0.813 |
| 7 | 0.990 | 0.111 |
| 8 | 0.995 | 0.529 |
| 9 | 0.532 | 0.319 |
| 10 | 0.981 | 0.803 |
| 11 | 0.731** | 0.742 |
| 12 | 0.932 | 0.209 |
| 13 | 0.970 | 0.710 |
| 14 | 0.984 | 0.606 |
| 15 | -0.487** | 0.231 |
| 16 | 0.467** | 0.736 |
| 17 | 0.901** | 0.969 |
| 18 | 0.478 | 0.327 |
| 19 | -0.756** | 0.691 |
| 20 | 0.460** | 0.552 |
| 21 | 0.584 | 0.490 |
| 22 | 0.561 | 0.343 |
| 23 | -0.514** | 0.486 |
| 24 | 0.726** | 0.950 |
| 25 | 0.817 | 0.477 |
| 26 | 0.282** | 0.467 |
| 27 | 0.562 | -0.725 |
| 28 | 0.880 | 0.827 |
| 29 | 0.284** | 0.573 |
| 30 | -1.000** | 0.877 |
| 31 | 0.996 | 0.569 |
| 32 | 0.773 | -0.000 |

** Significant differences

Overall Fit Indices Per Component

| Component | Observed Congruence | Critical value at alpha = 0.05 |
|---|---|---|
| 1 | 0.727 | 0.683 |
| 2 | 0.179** | 0.638 |

** Significant differences

Overall Fit Index

| Observed Congruence | Critical value at alpha = 0.05 |
|---|---|
| 0.438** | 0.694 |

** Significant differences

DISCREPANCY INDICES

Overall Fit Indices Per Item

| Item | Observed Similitude | Critical value at alpha = 0.05 |
|---|---|---|
| 1 | 0.190 | 0.587 |
| 2 | 0.215 | 0.358 |
| 3 | 0.306 | 0.563 |
| 4 | 0.003 | 0.636 |
| 5 | 3.730** | 0.272 |
| 6 | 0.763 | 0.823 |
| 7 | 0.188 | 0.425 |
| 8 | 0.023 | 0.602 |
| 9 | 0.411 | 0.509 |
| 10 | 0.178 | 0.347 |
| 11 | 0.595 | 0.860 |
| 12 | 0.176 | 1.471 |
| 13 | 0.323 | 0.402 |
| 14 | 0.054 | 0.339 |
| 15 | 1.130 | 1.567 |
| 16 | 0.788 | 0.965 |
| 17 | 3.506** | 0.930 |
| 18 | 0.917 | 1.435 |
| 19 | 1.893** | 1.001 |
| 20 | 0.945 | 1.174 |
| 21 | 0.691 | 0.819 |
| 22 | 0.823 | 0.888 |
| 23 | 2.860** | 1.180 |
| 24 | 1.774** | 1.324 |
| 25 | 1.135 | 2.234 |
| 26 | 1.176 | 1.762 |
| 27 | 1.269 | 1.799 |
| 28 | 0.708 | 1.052 |
| 29 | 0.801** | 0.794 |
| 30 | 1.683** | 1.381 |
| 31 | 0.031 | 0.956 |
| 32 | 0.521 | 1.320 |

** Significant differences

Overall Fit Indices Per Component

| Component | Observed Discrepancy | Critical value at alpha = 0.05 |
|---|---|---|
| 1 | 6.510 | 12.905 |
| 2 | 23.294** | 14.060 |

** Significant differences

Overall Fit Index

| Observed Discrepancy | Critical value at alpha = 0.05 |
|---|---|
| 29.804** | 21.728 |

** Significant differences

---

IMINCE completed
Computing time: 1.05000000 minutes.

**IMINCE Output: German and Kenyan sample (after omitting items)**

I M I N C E

Item Measurement Invariance

Based on Exploratory Factor Analysis

Release Version 2.01

August, 2002

University Rovira i Virgili

Tarragona, SPAIN

Programing:

Urbano Lorenzo-Seva

Mathematical Specification:

Urbano Lorenzo-Seva

Pere J. Ferrando

-------------------------------------------------------------------------------

DETAILS OF ANALYSIS

Target sample data file : German sample

Number of observations in Target sample : 206

Replication sample data file : Kenyan sample

Number of observations in Replication sample : 91

Number of variables : 10

Number of components : 2

Dispersion matrix : Covariances

Method for components extraction : Principal Components Analysis

Rotation to simplicity of Target loading matrix : Normalized Varimax

Rotation to similitude between loading matrices : Orthogonal Procrustes

Bias corrected Percentile intervals obtained by : Bootstrap resampling

Critical values obtained by : Bootstrap resampling

Number of samples in Bootstrap resampling : 5000

Alpha value : 0.05

-------------------------------------------------------------------------------

ITEM DIFFICULTIES

| Item | Target Sample | Replication Sample | Student's t | Effect Size (Cohen's d) |
|---|---|---|---|---|
| 1 | 4.16 | 3.82 | 1.55 | 0.20 |
| 2 | 3.19 | 3.24 | -0.25 | -0.03 |
| 3 | 2.39 | 2.65 | -1.20 | -0.15 |
| 4 | 3.36 | 3.14 | 0.68 | 0.09 |
| 5 | 3.09 | 2.54 | 2.56** | 0.32 |
| 6 | 4.38 | 4.81 | -1.99** | -0.25 |
| 7 | 4.23 | 4.37 | -0.67 | -0.08 |
| 8 | 3.24 | 2.79 | 2.11** | 0.27 |
| 9 | 3.02 | 2.43 | 2.76** | 0.35 |
| 10 | 4.46 | 4.78 | -1.71 | -0.22 |

**Significant differences

-------------------------------------------------------------------------------

ITEM DISCRIMINATIONS

Item discriminations (Loading Matrix)

Target Sample

| Item | C 1 | C 2 |
|---|---|---|
| 1 | 0.019 | 0.480 |
| 2 | -0.260 | 0.012 |
| 3 | 0.975 | -0.183 |
| 4 | 0.403 | 2.712 |
| 5 | 1.236 | 0.295 |
| 6 | 1.239 | 0.422 |
| 7 | 1.268 | 0.481 |
| 8 | 0.769 | 0.404 |
| 9 | 1.005 | -0.061 |
| 10 | 0.888 | 0.237 |

Bias-corrected Percentile Intervals of Item discriminations

Target Sample

| Item | C 1 | C 2 |
|---|---|---|
| 1 | ( -1.203; 1.744) | ( 0.110; 1.743) |
| 2 | ( -1.335; 1.243) | ( -0.573; 0.518) |
| 3 | ( -0.433; 1.733) | ( -0.707; 0.171) |
| 4 | ( -0.220; 0.962) | ( 1.763; 3.521) |
| 5 | ( -0.002; 1.800) | ( -0.192; 0.939) |
| 6 | ( -0.128; 1.898) | ( -0.095; 1.210) |
| 7 | ( -0.152; 1.943) | ( -0.033; 1.255) |
| 8 | ( -0.490; 1.515) | ( -0.122; 1.493) |
| 9 | ( -0.122; 1.743) | ( -0.379; 0.312) |
| 10 | ( -0.027; 1.779) | ( -0.065; 0.728) |

Item discriminations (Loading Matrix)

Replication Sample

| Item | C 1 | C 2 |
|---|---|---|
| 1 | -0.475 | 1.170 |
| 2 | -0.243 | 0.499 |
| 3 | 1.408 | 0.132 |
| 4 | 0.659 | 1.583 |
| 5 | 1.380 | 0.210 |
| 6 | 1.338 | 0.971 |
| 7 | 1.424 | 0.837 |
| 8 | 1.510 | 0.146 |
| 9 | 1.633 | -0.239 |
| 10 | 1.359 | 0.822 |

Bias-corrected Percentile Intervals of Item discriminations

Replication Sample

| Item | C 1 | C 2 |
|---|---|---|
| 1 | ( -0.874; 0.372) | ( 0.384; 1.545) |
| 2 | ( -0.929; 1.029) | ( -1.063; 1.188) |
| 3 | ( 0.259; 2.473) | ( -0.496; 0.818) |
| 4 | ( -0.932; 1.811) | ( 0.792; 2.215) |
| 5 | ( 0.178; 2.414) | ( -0.426; 0.975) |
| 6 | ( 0.088; 2.557) | ( 0.363; 1.585) |
| 7 | ( 0.288; 2.574) | ( 0.294; 1.459) |
| 8 | ( 0.557; 2.549) | ( -0.452; 0.799) |
| 9 | ( 0.576; 2.648) | ( -0.825; 0.393) |
| 10 | ( 0.069; 2.593) | ( 0.220; 1.475) |

Difference Values of Item discriminations

| Item | C 1 | C 2 |
|---|---|---|
| 1 | 0.495 | -0.690 |
| 2 | -0.017 | -0.488 |
| 3 | -0.433 | -0.316 |
| 4 | -0.255 | 1.128 |
| 5 | -0.144 | 0.086 |
| 6 | -0.099 | -0.549 |
| 7 | -0.156 | -0.355 |
| 8 | -0.741 | 0.257 |
| 9 | -0.628 | 0.178 |
| 10 | -0.472 | -0.585 |

** Significant differences

-----------------------------------------------------------------------------

ITEM RESIDUAL VARIANCES

Residual Variances

Target Sample

| Item | Residual Variance |
|---|---|
| 1 | 2.541 |
| 2 | 2.658 |
| 3 | 1.546 |
| 4 | 0.221 |
| 5 | 1.037 |
| 6 | 0.920 |
| 7 | 0.783 |
| 8 | 1.769 |
| 9 | 1.443 |
| 10 | 0.899 |

Bias-corrected Percentile Intervals of Residual Variances

Target Sample

| Item | Residual Variance Interval |
|---|---|
| 1 | ( 0.426; 3.524) |
| 2 | ( 0.284; 3.571) |
| 3 | ( 0.736; 2.513) |
| 4 | ( 0.019; 1.729) |
| 5 | ( 0.749; 1.591) |
| 6 | ( 0.521; 1.327) |
| 7 | ( 0.439; 1.134) |
| 8 | ( 0.854; 2.563) |
| 9 | ( 1.049; 1.901) |
| 10 | ( 0.581; 1.231) |

Residual Variances

Replication Sample

| Item | Residual Variance |
|---|---|
| 1 | 1.517 |
| 2 | 2.754 |
| 3 | 1.524 |
| 4 | 1.292 |
| 5 | 1.531 |
| 6 | 0.912 |
| 7 | 0.934 |
| 8 | 1.446 |
| 9 | 1.192 |
| 10 | 0.922 |

Bias-corrected Percentile Intervals of Residual Variances

Replication Sample

| Item | Residual Variance Interval |
|---|---|
| 1 | ( 0.857; 2.866) |
| 2 | ( 2.068; 3.798) |
| 3 | ( 1.145; 2.029) |
| 4 | ( 0.875; 2.485) |
| 5 | ( 1.056; 2.060) |
| 6 | ( 0.612; 1.314) |
| 7 | ( 0.633; 1.509) |
| 8 | ( 0.994; 2.088) |
| 9 | ( 0.767; 1.772) |
| 10 | ( 0.660; 1.344) |

Difference Values in Residual Variances

| Item | Residual Variance |
|---|---|
| 1 | 1.024 |
| 2 | -0.095 |
| 3 | 0.021 |
| 4 | -1.071 |
| 5 | -0.494 |
| 6 | 0.008 |
| 7 | -0.151 |
| 8 | 0.322 |
| 9 | 0.251 |
| 10 | -0.023 |

** Significant differences

--------------------------------------------------------------------------------

OVERALL INDICES OF ITEM MEASUREMENT INVARIANCE

ITEM DIFFICULTIES

Hotteling's T Square

TSQ = 34.937440; Freedom degrees = 10 and 286; F = 3.387155 (P = 0.00000)

--------------------------------------------------------------------------------

OVERALL INDICES OF ITEM MEASUREMENT INVARIANCE

CONGRUENCE INDICES

Overall Fit Indices Per Item

| Item | Observed Congruence | Critical value at alpha = 0.05 |
|---|---|---|
| 1 | 0.910 | 0.477 |
| 2 | 0.478 | -0.969 |
| 3 | 0.961 | 0.160 |
| 4 | 0.970 | 0.904 |
| 5 | 0.997 | -0.109 |
| 6 | 0.955 | -0.247 |
| 7 | 0.986 | 0.216 |
| 8 | 0.926 | -0.667 |
| 9 | 0.996 | 0.120 |
| 10 | 0.960 | -0.828 |

** Significant differences

Overall Fit Indices Per Component

| Component | Observed Congruence | Critical value at alpha = 0.05 |
|---|---|---|
| 1 | 0.971 | 0.206 |
| 2 | 0.807 | 0.441 |

** Significant differences

Overall Fit Index

| Observed Congruence | Critical value at alpha = 0.05 |
|---|---|
| 0.886 | 0.551 |

** Significant differences

DISCREPANCY INDICES

Overall Fit Indices Per Item

| Item | Observed Similitude | Critical value at alpha = 0.05 |
|---|---|---|
| 1 | 0.721 | 1.697 |
| 2 | 0.238 | 3.662 |
| 3 | 0.287 | 2.090 |
| 4 | 1.338 | 2.087 |
| 5 | 0.028 | 2.504 |
| 6 | 0.312 | 2.889 |
| 7 | 0.151 | 2.710 |
| 8 | 0.615 | 2.050 |
| 9 | 0.426 | 1.853 |
| 10 | 0.565 | 2.282 |

** Significant differences

Overall Fit Indices Per Component

| Component | Observed Discrepancy | Critical value at alpha = 0.05 |
| --- | --- | --- |
| 1 | 1.719 | 14.121 |
| 2 | 2.962 | 8.825 |

** Significant differences

Overall Fit Index

| Observed Discrepancy | Critical value at alpha = 0.05 |
| --- | --- |
| 4.681 | 19.002 |

** Significant differences

--------------------------------------------------------------------------------

IMINCE completed

Computing time: 0.25000000 minutes.

## IMINCE Output: German and Polish sample (after omitting items)

I M I N C E

Item Measurement Invariance
Based on Exploratory Factor Analysis

Release Version 2.01
August, 2002
University Rovira i Virgili
Tarragona, SPAIN

Programing:
Urbano Lorenzo-Seva

Mathematical Specification:
Urbano Lorenzo-Seva
Pere J. Ferrando

------------------------------------------------------------------------------

DETAILS OF ANALYSIS

Target sample data file : German sample
Number of observations in Target sample : 206
Replication sample data file : Polish sample
Number of observations in Replication sample : 108

Number of variables : 11
Number of components : 2

Dispersion matrix : Covariances
Method for components extraction : Principal Components Analysis

Rotation to simplicity of Target loading matrix : Normalized Varimax
Rotation to similitude between loading matrices : Orthogonal Procrustes

Bias corrected Percentile intervals obtained by : Bootstrap resampling
Critical values obtained by : Bootstrap resampling
Number of samples in Bootstrap resampling : 5000

Alpha value : 0.05

------------------------------------------------------------------------------

ITEM DIFFICULTIES

| Item | Target Sample | Replication Sample | Student's t | Effect Size (Cohen's d) |
|---|---|---|---|---|
| 1 | 3.52 | 3.24 | 1.66 | 0.20 |
| 2 | 3.53 | 2.91 | 3.09** | 0.37 |
| 3 | 2.77 | 3.10 | -1.93 | -0.23 |
| 4 | 3.98 | 3.89 | 0.51 | 0.06 |
| 5 | 2.97 | 2.94 | 0.21 | 0.02 |
| 6 | 3.32 | 2.86 | 3.03** | 0.36 |
| 7 | 2.72 | 3.36 | -3.40** | -0.40 |
| 8 | 4.48 | 4.71 | -1.39 | -0.17 |
| 9 | 4.23 | 4.41 | -0.93 | -0.11 |

| | | | | |
|---|---|---|---|---|
| 10 | 3.02 | 3.64 | -3.09** | -0.37 |
| 11 | 4.46 | 4.44 | 0.08 | 0.01 |

**Significant differences

-------------------------------------------------------------------------------

ITEM DISCRIMINATIONS

Item discriminations (Loading Matrix)
Target Sample

| Item | C 1 | C 2 |
|---|---|---|
| 1 | 0.684 | 0.062 |
| 2 | -1.038 | 0.341 |
| 3 | 0.592 | -0.108 |
| 4 | -0.652 | 1.070 |
| 5 | 0.814 | -0.167 |
| 6 | 0.618 | -0.166 |
| 7 | 1.091 | 0.518 |
| 8 | -0.234 | 1.369 |
| 9 | -0.001 | 1.439 |
| 10 | 0.897 | 0.783 |
| 11 | 0.050 | 1.059 |

Bias-corrected Percentile Intervals of Item discriminations
Target Sample

| Item | C 1 | C 2 |
|---|---|---|
| 1 | ( 0.381; 1.344) | ( -0.154; 1.204) |
| 2 | ( -2.312; -0.521) | ( -0.447; 1.989) |
| 3 | ( 0.301; 1.387) | ( -0.348; 1.068) |
| 4 | ( -0.942; -0.308) | ( 0.173; 1.922) |
| 5 | ( 0.496; 1.586) | ( -1.015; 0.387) |
| 6 | ( 0.398; 0.837) | ( -0.380; 0.184) |
| 7 | ( 0.769; 1.485) | ( -0.613; 1.333) |
| 8 | ( -0.494; 0.033) | ( 0.078; 2.085) |
| 9 | ( -0.294; 0.223) | ( 0.162; 2.106) |
| 10 | ( 0.603; 1.190) | ( -0.211; 1.551) |
| 11 | ( -0.225; 0.352) | ( 0.157; 1.858) |

Item discriminations (Loading Matrix)
Replication Sample

| Item | C 1 | C 2 |
|---|---|---|
| 1 | 0.796 | -0.155 |
| 2 | -0.688 | 0.271 |
| 3 | 0.986 | -0.230 |
| 4 | -0.818 | 0.577 |
| 5 | 1.051 | -0.508 |
| 6 | 0.563 | -0.477 |
| 7 | 0.979 | -0.204 |
| 8 | -0.159 | 0.417 |
| 9 | -0.115 | 0.980 |
| 10 | 0.928 | 1.333 |
| 11 | -0.109 | 0.671 |

Bias-corrected Percentile Intervals of Item discriminations
Replication Sample

| Item | C 1 | C 2 |
|---|---|---|
| 1 | ( 0.485; 1.056) | ( -0.442; 0.415) |
| 2 | ( -1.064; -0.261) | ( -0.560; 0.611) |
| 3 | ( 0.582; 1.225) | ( -0.773; 0.272) |
| 4 | ( -1.078; -0.467) | ( 0.263; 1.002) |
| 5 | ( 0.811; 1.259) | ( -0.837; -0.139) |
| 6 | ( 0.235; 0.805) | ( -0.775; -0.065) |
| 7 | ( 0.675; 1.225) | ( -0.630; 0.250) |
| 8 | ( -0.474; 0.119) | ( 0.142; 0.851) |
| 9 | ( -0.667; 0.813) | ( 0.023; 2.606) |
| 10 | ( 0.474; 1.305) | ( 0.213; 2.540) |
| 11 | ( -0.492; 0.274) | ( 0.040; 1.019) |

Difference Values of Item discriminations

| Item | C 1 | C 2 |
|---|---|---|
| 1 | -0.112 | 0.217 |
| 2 | -0.349 | 0.070 |
| 3 | -0.394 | 0.122 |
| 4 | 0.165 | 0.492 |
| 5 | -0.237 | 0.341 |
| 6 | 0.055 | 0.311 |
| 7 | 0.112 | 0.722 |
| 8 | -0.075 | 0.952 |
| 9 | 0.114 | 0.460 |
| 10 | -0.031 | -0.550 |
| 11 | 0.159 | 0.388 |

** Significant differences

--------------------------------------------------------------------------------

ITEM RESIDUAL VARIANCES

Residual Variances
Target Sample

| Item | Residual Variance |
|---|---|
| 1 | 1.710 |
| 2 | 2.143 |
| 3 | 1.717 |
| 4 | 0.857 |
| 5 | 1.426 |
| 6 | 1.224 |
| 7 | 1.197 |
| 8 | 0.592 |
| 9 | 0.551 |
| 10 | 1.037 |
| 11 | 0.619 |

Bias-corrected Percentile Intervals of Residual Variances
Target Sample

| Item | Residual Variance Interval |
|---|---|
| 1 | ( 0.524; 2.649) |
| 2 | ( 0.098; 2.920) |
| 3 | ( 0.847; 2.607) |
| 4 | ( 0.553; 1.263) |
| 5 | ( 0.712; 2.111) |

| | | |
|---|---|---|
| 6 | ( 0.972; | 1.497) |
| 7 | ( 0.794; | 1.733) |
| 8 | ( 0.291; | 1.116) |
| 9 | ( 0.282; | 1.424) |
| 10 | ( 0.804; | 1.481) |
| 11 | ( 0.361; | 1.016) |

Residual Variances
Replication Sample

| Item | Residual Variance |
|---|---|
| 1 | 0.988 |
| 2 | 1.574 |
| 3 | 1.030 |
| 4 | 1.078 |
| 5 | 0.643 |
| 6 | 0.983 |
| 7 | 1.174 |
| 8 | 0.709 |
| 9 | 1.657 |
| 10 | 0.943 |
| 11 | 0.804 |

Bias-corrected Percentile Intervals of Residual Variances
Replication Sample

| Item | Residual Variance Interval | |
|---|---|---|
| 1 | ( 0.725; | 1.435) |
| 2 | ( 1.168; | 2.235) |
| 3 | ( 0.801; | 1.693) |
| 4 | ( 0.746; | 1.564) |
| 5 | ( 0.519; | 1.307) |
| 6 | ( 0.737; | 1.374) |
| 7 | ( 0.868; | 1.770) |
| 8 | ( 0.494; | 1.112) |
| 9 | ( 0.622; | 4.240) |
| 10 | ( 0.027; | 3.014) |
| 11 | ( 0.409; | 1.406) |

Difference Values in Residual Variances

| Item | Residual Variance |
|---|---|
| 1 | 0.722 |
| 2 | 0.569 |
| 3 | 0.687 |
| 4 | -0.221 |
| 5 | 0.783 |
| 6 | 0.242 |
| 7 | 0.022 |
| 8 | -0.117 |
| 9 | -1.106 |
| 10 | 0.094 |
| 11 | -0.185 |

** Significant differences

-----------------------------------------------------------------------------

OVERALL INDICES OF ITEM MEASUREMENT INVARIANCE

ITEM DIFFICULTIES

Hotteling's T Square

TSQ = 42.508954; Freedom degrees = 11 and 302; F = 3.740590 (P = 0.00000)

-------------------------------------------------------------------------------

OVERALL INDICES OF ITEM MEASUREMENT INVARIANCE

CONGRUENCE INDICES

Overall Fit Indices Per Item

| Item | Observed Congruence | Critical value at alpha = 0.05 |
|---|---|---|
| 1 | 0.960 | 0.816 |
| 2 | 0.998 | 0.875 |
| 3 | 0.999 | 0.769 |
| 4 | 0.918 | 0.780 |
| 5 | 0.969 | 0.908 |
| 6 | 0.904 | 0.896 |
| 7 | 0.797 | 0.770 |
| 8 | 0.981 | 0.782 |
| 9 | 0.993 | 0.938 |
| 10 | 0.970 | 0.820 |
| 11 | 0.978 | 0.816 |

** Significant differences

Overall Fit Indices Per Component

| Component | Observed Congruence | Critical value at alpha = 0.05 |
|---|---|---|
| 1 | 0.964 | 0.659 |
| 2 | 0.801 | 0.732 |

** Significant differences

Overall Fit Index

| Observed Congruence | Critical value at alpha = 0.05 |
|---|---|
| 0.872 | 0.713 |

** Significant differences

DISCREPANCY INDICES

Overall Fit Indices Per Item

| Item | Observed Similitude | Critical value at alpha = 0.05 |
|---|---|---|
| 1 | 0.060 | 0.834 |
| 2 | 0.127 | 0.949 |

| | | |
|---|---|---|
| 3 | 0.170 | 0.529 |
| 4 | 0.270 | 0.842 |
| 5 | 0.173 | 0.341 |
| 6 | 0.100 | 0.137 |
| 7 | 0.534 | 0.850 |
| 8 | 0.912 | 1.206 |
| 9 | 0.224 | 2.517 |
| 10 | 0.304 | 0.773 |
| 11 | 0.176 | 0.852 |

** Significant differences

Overall Fit Indices Per Component

| Component | Observed Discrepancy | Critical value at alpha = 0.05 |
|---|---|---|
| 1 | 0.434 | 5.251 |
| 2 | 2.615 | 4.352 |

** Significant differences

Overall Fit Index

| Observed Discrepancy | Critical value at alpha = 0.05 |
|---|---|
| 3.048 | 7.967 |

** Significant differences

--------------------------------------------------------------------------------

IMINCE completed
Computing time: 0.31666667 minutes.
